# Microtubule-associated protein IQ67 DOMAIN5 regulates morphogenesis of leaf pavement cells in *Arabidopsis thaliana*

**DOI:** 10.1093/jxb/ery395

**Published:** 2018-11-08

**Authors:** Dipannita Mitra, Sandra Klemm, Pratibha Kumari, Jakob Quegwer, Birgit Möller, Yvonne Poeschl, Paul Pflug, Gina Stamm, Steffen Abel, Katharina Bürstenbinder

**Affiliations:** 1 Department of Molecular Signal Processing, Leibniz Institute of Plant Biochemistry (IPB),Halle (Saale), Germany; 2 Institute of Computer Science, Martin Luther University Halle-Wittenberg, Halle (Saale), Germany; 3 iDiv, German Integrative Research Center for Biodiversity, Leipzig, Germany; 4 Institute of Biochemistry and Biotechnology, Martin Luther University Halle-Wittenberg, Halle (Saale), Germany; 5 Department of Plant Sciences, University of California, Davis, CA, USA

**Keywords:** Arabidopsis, calcium, calmodulin, cell wall, IQ67 DOMAIN, microtubules, pavement cell shape, signaling

## Abstract

Plant microtubules form a highly dynamic intracellular network with important roles for regulating cell division, cell proliferation, and cell morphology. Their organization and dynamics are co-ordinated by various microtubule-associated proteins (MAPs) that integrate environmental and developmental stimuli to fine-tune and adjust cytoskeletal arrays. IQ67 DOMAIN (IQD) proteins recently emerged as a class of plant-specific MAPs with largely unknown functions. Here, using a reverse genetics approach, we characterize Arabidopsis IQD5 in terms of its expression domains, subcellular localization, and biological functions. We show that *IQD5* is expressed mostly in vegetative tissues, where it localizes to cortical microtubule arrays. Our phenotypic analysis of *iqd5* loss-of-function lines reveals functions of IQD5 in pavement cell (PC) shape morphogenesis. Histochemical analysis of cell wall composition further suggests reduced rates of cellulose deposition in anticlinal cell walls, which correlate with reduced anisotropic expansion. Lastly, we demonstrate IQD5-dependent recruitment of calmodulin calcium sensors to cortical microtubule arrays and provide first evidence for important roles for calcium in regulation of PC morphogenesis. Our work identifies IQD5 as a novel player in PC shape regulation and, for the first time, links calcium signaling to developmental processes that regulate anisotropic growth in PCs.

## Teaser

Microtubule-localized Arabidopsis IQ67 DOMAIN5 regulates pavement cell morphogenesis in the leaf epidermis and links calcium–calmodulin signaling to anisotropic expansion during early phases of pavement cell growth.

## Introduction

The plant cytoskeleton, comprised of actin filaments and microtubules (MTs), forms a three-dimensional intracellular network that determines cell division and cell morphology, and serves as tracks for cellular transport of various cargoes, including organelles, proteins, and other macromolecular complexes (Wasteneys and Yang, 2004*b*; Hussey *et al.*, 2006; Akhmanova and Hammer, 2010). Networks of MTs form highly dynamic arrays and adopt specific functions during the plant life cycle, including essential roles in cell division and expansion, intra- and intercellular transport, cellular organization, and the deposition of cell wall material. In interphase cells, MTs reorganize into cortical networks tethered to the plasma membrane (PM), which serve as tracks for PM-localized cellulose synthase complexes (CSCs) and thereby define the direction of cellulose deposition (Paredez *et al.*, 2006; Liu *et al.*, 2015). In addition to their important roles in development, MT arrays function during growth adaptation in response to changing environmental conditions, thereby contributing to plant fitness (Wasteneys and Yang, 2004*a*).

To engage in these diverse cellular functions, MT organization and dynamics are tightly controlled (Wasteneys, 2002). Developmental and environmental stimuli can induce rapid reorganization of the MT cytoskeleton (e.g. in response to mechanical stimulation) which can occur within a few minutes and involves changes in MT trajectories, as well as altered rates of (de-) polymerization (Hardham *et al.*, 2008). Phytohormones exert control over MT orientation (Shibaoka, 1994; Locascio *et al.*, 2013; Takatani *et al.*, 2015), and signaling via the second messenger calcium (Ca^2+^) has been implicated in cytoskeletal control, as suggested by sensitivity of MT stability to elevated Ca^2+^ concentrations (Hepler, 2005, 2016). MT-associated proteins (MAPs), which bind to tubulin subunits, play essential roles for regulating cytoskeletal behavior (Lloyd and Hussey, 2001; Sedbrook, 2004) and are likely candidates to integrate incoming signals into appropriate responses. Numerous MAPs have been identified in plants, which mediate bundling, cross-linking, nucleation, or severing of MTs, or, in the case of plus end-tracking MAPs, control dynamic instability at polymerizing plus ends (Akhmanova and Steinmetz, 2008; Horio and Murata, 2014). Other MAPs facilitate physical connections between MTs and protein complexes, such as CSCs (Bringmann *et al.*, 2012), or cross-linking to the actin cytoskeleton (Schneider and Persson, 2015). MAPs also mediate tethering of MTs to the PM (Bayer *et al.*, 2017; Oda, 2018), which is required for stabilization against the pushing forces of CSCs (Liu *et al.*, 2016) and potentially contributes to subcompartmentalization of PMs into functional subdomains (Sugiyama *et al.*, 2017). Still, the modes by which external signals are integrated into MT (re-) orientation and how MAPs contribute to it are poorly understood.

We previously identified IQ67 DOMAIN (IQD) family proteins as the largest known class of MAPs in plants (Bürstenbinder *et al.*, 2017*b*), which are encoded by multigene families of 23–66 members in several angiosperms, including *Arabidopsis thaliana*, *Oryza sativa* (rice), *Solanum lycopersicum* (tomato), and *Glycine max* (Abel *et al.*, 2005; Huang *et al.*, 2013; Feng *et al.*, 2014). The family-defining IQ67 domain harbors motifs with predicted roles in binding to calmodulin (CaM) Ca^2+^ sensor proteins that are an integral part of the cellular Ca^2+^ decoding machinery (Abel *et al.*, 2005). Thus, IQDs are likely candidates for integration of CaM-dependent Ca^2+^ signaling into MT (re-)organization and growth regulation (Bürstenbinder *et al.*, 2017*a*). First experimental data point to important roles for IQD proteins in plant development, as indicated by altered fruit shape and grain size in plants with elevated expression levels of tomato *SUN/IQD12* and rice *GRAIN SIZE ON CHROMOSOME 5(GSE5)/IQD21*, respectively (Xiao *et al.*, 2008; Duan *et al.*, 2017). Functions of IQDs in MT organization are supported by differential MT patterns, which are induced upon overexpression of individual family members in transient expression assays in *Nicotiana benthamiana* (Bürstenbinder *et al.*, 2017*b*). Divergent MT patterns in *N. benthamiana* correlate with divergent effects on MT organization and growth in transgenic Arabidopsis *IQD* overexpression plants, as indicated by altered plant morphology and cell shape upon ectopic overexpression of, for example, *IQD14* and *IQD16* visible during development and in diverse tissues (Bürstenbinder *et al.*, 2017*b*). Mechanistic studies on IQD functions, however, are still limited because (i) phenotypes are almost exclusively reported in *IQD* gain-of-function or overexpression lines; (ii) multigene families are not easily amenable to reverse genetics approaches due to functional redundancies; and (iii) insights into the spatial and temporal control of Ca^2+^ signal generation during development are sparse due to limited sensitivities of intracellular Ca^2+^ imaging methods (Kudla *et al.*, 2018).

To identify functions of family members, we selected Arabidopsis IQD5, because MT pattern analysis upon overexpression of *YFP-IQD5* in transient expression assays in *N. benthamiana* suggested unique and specific roles for this family member in MT organization (Bürstenbinder *et al.*, 2017*b*). Moreover, IQD5, which belongs to phylogenetic subgroup IIIa of the IQD family, clusters separately from the other members of this subgroup, namely IQD6–IQD10 (Abel *et al.*, 2005), and thus may have specialized functions *in planta*. In this study, we present a systematic analysis of Arabidopsis *IQD5* using reverse genetics approaches. We identified expression domains of *IQD5* by analysis of transgenic *pIQD5::GFP-GUS* reporter lines, and determined its subcellular localization in transgenic *pIQD5::IQD5-GFP/iqd5-1* lines. We show that IQD5–green fluorescent protein (GFP) decorates cortical MTs at neck regions of leaf epidermis pavement cells (PCs). Loss of *IQD5* results in strongly reduced growth restriction at neck regions, which correlates with a reduced deposition of cellulose in anticlinal walls of PCs. Recombinant IQD5 interacts with apo-CaM and Ca^2+^–CaM *in vitro*, and IQD5 recruits CaM to MTs *in planta*. Moreover, we show that PC shape is sensitive to elevated external Ca^2+^ concentrations. Together, our research provides evidence for functions of IQD5 in shape establishment of leaf epidermis PCs and for the first time links Ca^2+^ signaling to the control of interdigitated growth of PCs.

## Materials and methods

### Plant material, growth conditions, and macroscopic phenotyping

Wild-type (WT) seeds (Col-0 accession) were originally obtained from the Arabidopsis Biological Resource Center. T-DNA insertion lines SALK_015580 and GK-288E09, referred to as *iqd5-1* and *iqd5-2*, respectively, were obtained from the Nottingham Arabidopsis Stock Centre. Genomic DNA was extracted as described in Bürstenbinder *et al.* (2007). Homozygous mutant lines were identified by PCR-based genotyping with the following primer combinations: *iqd5-1*, WT allele IQD105 (5'-GATTATCTCTGCCAAACAGCG-3') and IQD106 (5'-GGAGAGTGACTTGGGCTGAC-3'), insert IQD105+A004 (5'-ATTTTGCCGATTTCGGAAC-3'); *iqd5-2*, WT allele IQD075 (5'-ATGGGAGCTTCAGGGAGATG-3')+IQD076 (5'-GCGTTACAGCAGCTTGTTTTC-3'), and insert IQD076+A009 (5'-ATAATAACGCTGCGGACATCTACATTT-3'). A 2201 bp (*pIQD5*_*long*_) and a 1207 bp (*pIQD5*_*short*_) fragment of the *IQD5* promoter sequence were amplified from genomic DNA with IQD180 (5'-CACCTCTATATATGGTTCACAATCGAGACAC-3') and IQD345 (5'-CACCATAAATCACATCACTGTTTTTGGGT-3') forward primers, respectively, in combination with the IQD181 reverse primer (5'-TCTATCTCAATTCCAACGATCAG-3'), and mobilized into the pENTR/dTOPO vector. A genomic *pIQD5*_*short*_*::IQD5(w/-stop codon*) fragment was amplified with forward primer IQD1521 (5'-attB1-TCTCTATATATGGTTCACAATCGAGACAC-3') and reverse primer IQD1522 (5'-attB2-CTGCAAGCCTCTGTTTTATTGGGTCGG-3'), and mobilized into pDONR221. Fidelity of inserts was verified by sequencing. For generation of transgenic *pIQD5::GFP-GUS* and *pIQD5::IQD5-GFP* lines, the inserts were mobilized into pBGWFS7 and pB7FWG,0, respectively (Karimi *et al.*, 2002). Arabidopsis plants were transformed by *Agrobacterium tumefaciens*-mediated transfection using the floral dip method (Clough and Bent, 1998). Per construct, 10–24 independent lines were identified in the T_1_ generation by Basta selection. T_2_ plants were screened for the presence of single-copy T-DNA insertion by segregation analysis (Basta). For analysis of GFP fluorescence and β-glucuronidase (GUS) expression, 2–4 homozygous T_3_ lines were included, which showed representative GFP fluorescence or *GUS* expression patterns.

Seeds were surface sterilized with chlorine gas, stratified for 2 d at 4 °C on *Arabidopsis thaliana* Salts (ATS) medium [1× ATS, 0.5% (w/v) agar gel, 1% (w/v) sucrose] (Lincoln *et al.*, 1990), and grown at 21°C under long-day conditions (16 h light, 8 h dark). For oryzalin treatments, seedlings were incubated for 1–2 h in liquid medium supplemented with 10 µM oryzalin in a final concentration of 0.25% (v/v) DMSO or the DMSO control as described in Bürstenbinder *et al.* (2013). Macroscopic growth parameters were analyzed in 5-day-old seedling and in 3-week-old plants. Root length was quantified with RootDetection (http://www.labutils.de/rd.html, last accessed 19 November 2018). Cotyledon and leaf area were measured with the Easy Leaf Area software (http://www.plant-image-analysis.org/software/easy-leaf-area, last accessed 19 November 2018) according to the manual. For treatment with Ca^2+^, seedlings were grown for 5 d on half-strength Murashige and Skoog (1/2 MS) medium supplemented with the indicated concentrations of CaCl_2_ according to Chen *et al.* (2014).

### RNA extraction and expression analysis

Total RNA was extracted from 2-week-old plants using TRIreagent. Synthesis of cDNA via reverse transcription and RT–PCRs were performed according to Bürstenbinder *et al.* (2007) with the following primers: *IQD5*, primer IQD075 and IQD117 (5'-CTATGCAAGCCTCTGTTTTATTGG-3'); *ACTIN2*, primer A005 (5'-CAAAGACCAGCTCTTCCATC-3'); and A006 (5'-CTGTGAACGATTCCTGGACCT-3'). For quantitative real-time PCR (qRT-PCR), conditions were selected as described in Bürstenbinder *et al.* (2017*b*) and the following primers were used: *IQD5*, IQD1777 (CAACTAAAGCCAACCGAGCA-3') and IQD1778 (GGTTTTGGGCAGATTTTTCC-3'), *PP2A* A015 (AGCCAACTAGGACGGATCTGGT-3') and A016 (CTATCCGAACTTCTGCCTCATTA-3'). In brief, 3–5 shoots of 2-week-old plants were pooled for RNA extraction. A 2 µg aliquot of DNase I-treated RNA was reverse transcribed with oligo(dT) primers using the Revert Aid First Strand cDNA synthesis kit (Thermo Fisher) to generate first-strand cDNA. Primer efficiencies were calculated from standard curves. A 1 µl aliquot of 1:10-diluted cDNA was used in a 10 µl reaction mix including Fast SYBR Green master mix (Applied Biosystems), and qPCRs were run on a 7500 Fast Real-Time PCR system with the following program: 10 min, 95 °C; 40 cycles of 3 s, 95 °C, and 30 s, 63 °C. Expression levels of *IQD5* were calculated relative to *PP2A*.

### Microscopy, staining procedures, and image analysis

Whole-mount GUS staining of seedlings and plants was performed as described in Bürstenbinder *et al.* (2017*b*). Plant materials were cleared in chloral hydrate, and roots and seeds were imaged with a Zeiss axioplan 2 microscope using a differential interfernce contrast (DIC) objective. Imaging of whole seedlings, leaves, flowers, and siliques was performed with a Nikon SMZ 1270 stereo microscope.

Confocal imaging was performed with a Zeiss LSM 780 inverted microscope using a ×40 water immersion objective, unless stated otherwise. Generation of yellow fluorescent protein (YFP)–IQD5, Y_N_–TRM1, red fluorescent protein (RFP)–TUA5, and mCherry–CaM2 is described in Bürstenbinder *et al.* (2017*b*) and Gantner *et al.* (2018). GFP was excited using a 488 nm laser and emission was detected between 493 nm and 555 nm. YFP was excited by a 514 nm laser, and emission was detected between 525 nm and 550 nm. For mCherry excitation, a 555 nm laser was used, and emission was detected between 560 nm and 620 nm. Fluorescence intensities of IQD5–GFP adjacent to the periclinal wall at convex and concave sides of lobes were quantified according to Armour *et al.* (2015). Average fluorescence intensities were measured with FiJi (Schindelin *et al.*, 2012) in a total of six cells from three independent seedlings, and five lobes per cell were analyzed. The vector series of Gehl *et al.* (2009) was used for generation of the bimolecular fluorescence complementation (BiFC) construct. For all samples included in the BiFC experiment, imaging was performed with an identical laser setting. In co-expression assays, mCherry and GFP fluorescence were recorded in the sequential mode.

For visualization of cell contours, cell outlines were visualized by propidium iodide (PI) staining as described in Bürstenbinder *et al.* (2017*b*), and imaged with a ×20 objective [5–10 days after germination (DAG)] or with a ×40 objective (2 and 3 DAG). PI was excited with a 555 nm laser, and emission was detected between 560 nm and 620 nm. Segmentation, feature quantification, and graphical visualization of PC shapes were conducted with the ImageJ plugin PaCeQuant and the associated R script (Möller *et al.*, 2017). For cells in cotyledons 5–10 DAG and in the third true leaf, the threshold for size filtering implemented in PaCeQuant was set to the default value of 240 µm^2^. For cells in cotyledons 2 and 3 DAG, the threshold for size filtering was reduced to 75 µm^2^. For time series analysis of cells during cotyledon development, cells were grouped by their sizes into the following categories: tiny <240 µm^2^; small, 240–1400 µm^2^; medium, 1400–4042 µm^2^; and large >4042 µm^2^. Thresholds for small, medium, and large cells were chosen according to Möller *et al.* (2017).

For histochemical cellulose staining in cell walls, 5-day-old seedlings were incubated for 90 min in 0.04% (v/v) calcofluor white M2R dissolved in Tris–HCl buffer (pH 9.2). To stain callose and the cuticle, seedlings were incubated for 3 h and 5 min in 0.1% (v/v) aniline blue in 100 mM Na_2_PO_4_ buffer (pH 7.2) and 0.1% (w/v) auramine O in 50 mM Tris–HCl buffer (pH 7.2), respectively. Subsequently, seedlings were co-stained with PI to visualize cell contours. Dissected cotyledons were imaged with a Zeiss LSM 700 inverted microscope, using a ×40 water immersion objective. Calcofluor white, aniline blue, and auramine O were excited with a 405 nm laser, and emission was detected with a 490 nm short pass filter. Co-staining was recorded in the sequential mode.

To quantify fluorescence intensities along the boundaries of the cells, we established a workflow combining automatic segmentation based on the method implemented in PaCeQuant and quantification of fluorescence intensities along the contour segments. For each boundary pixel in an image, the set of adjacent cell regions in a 15 × 15 neighborhood around the pixel is determined and the fluorescence intensity value of the pixel is added to the total intensity sum of each of these regions. Finally, an average intensity value for the boundary of each cell region is calculated by dividing the intensity sum of the region through the total number of pixels that contributed to the specific region, which we implemented in MiToBo (Möller *et al.*, 2016).

### Structure prediction, protein expression, and calmodulin binding assays

Structural prediction of the IQ67 domain of IQD5 spanning amino acids E87–L153 was performed using PHYRE2 (Kelley *et al.*, 2015), which revealed the highest similarities with the crystal structures of the CaM-binding domains of mouse myosin V (PDB:2IX7, 99.5% similarity) (Houdusse *et al.*, 2006) and mouse myosin-1c (PDB:4R8G, 99.8%) (Lu *et al.*, 2015). The predicted structure of the IQ67 domain of IQD5 was aligned with PDB:2IX7, which contains the crystal structure of apo-CaM bound to the first two IQ motifs of myosin V, using PyMol (DeLano, 2009). CaM was fitted to adjust for the different spacing of IQ motifs by 11 and 12 amino acids in the CaM-binding domains of IQD5 and myosin V, respectively.

Expression of glutathione *S*-transferase (GST)–IQD5 and *in vitro* CaM binding assays were performed according to Levy *et al.* (2005). Generation of IQD5 pENTR vectors is described in Bürstenbinder *et al.* (2017*b*). The coding sequence (CDS) of IQD5 was mobilized into the pDEST15 vector (Invitrogen) to generate an N-terminal GST fusion, and GST–IQD5 and the GST control were expressed in the *Escherichia coli* strain KRX (Novagen) upon induction with 0.1% (w/v) rhamnose and 1 mM isopropyl-β-d-thiogalactopyranoside (IPTG). Cells were resuspended in CaM pull-down buffer [5.8 mM Tris–HCl, pH 7.3; 2.7 mM KCl; 127 mM NaCl; 0.1% (v/v) Tween 20; 0.002% (w/v) NaN_3_]. Bovine CaM immobilized on Sepharose beads (GE Healthcare) was incubated with cleared protein extracts in CaM buffer containing either 5 mM EGTA or 1 mM CaCl_2_. After four steps of washing, the last washing fraction and the bead fraction were collected, and, together with the unbound fraction, separated by SDS–PAGE. GST-tagged proteins were visualized by immunoblot analysis using a horseradish peroxidase (HRP)-coupled α-GST antibody (Santa Cruz).

### Statistical analysis

Statistical analysis of root length, cotyledon and leaf area, and *IQD5* expression was performed using ANOVA implemented in the R software, followed by a Tukey’s post-hoc test, and Benjamini–Hochberg adjustment of *P*-values. For statistical analysis of fluorescence intensities at convex and concave sides of pavement cells in *pIQD5::IQD5-GFP* seedlings, a *t*-test was performed. Statistical analysis of PC shape features was performed using the Kruskal–Wallis test, followed by a Dunn’s post-hoc test and Benjamini–Hochberg adjustment of *P*-values, which is part of the R script provided in the PaCeQuant package.

## Results

### *IQD5* is expressed in vegetative tissues

**Fig. 1.**
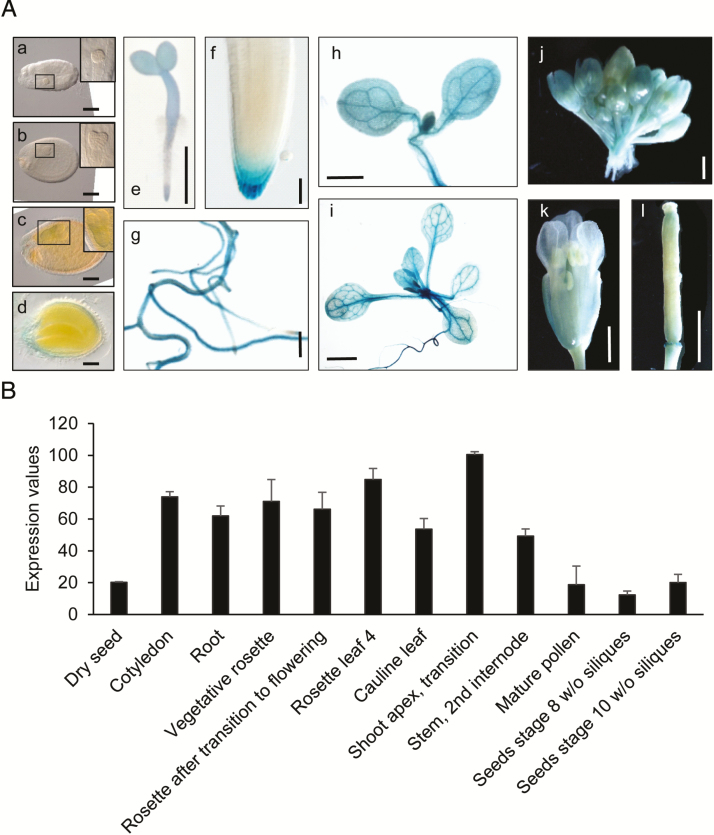
*IQD5* expression analysis. Whole-mount histochemical GUS staining of *ProIQD5*_*short*_*:GFP-GUS* reporter lines (A) in seeds and embryos of globular (a), heart (b), torpedo (c), and mature (d) stage, in 2-day-old seedlings (e); insets show close-ups of embryos, indicated by black boxes (a–c), in the primary root meristem (f), in lateral roots (g), and in cotyledons (h) of 5-day-old seedlings, in the shoot of 10-day-old seedlings (i), and in flower buds (j), flowers (k), and siliques (l) of 5-week-old plants. Scale bars represent 100 µm (a–d), 1 mm (e, g–l), and 10 µm (f). *In silico* expression data of *IQD5* in different tissues and organs were obtained from the publicly available eFP browser database (http://bar.utoronto.ca/efp/cgi-bin/efpWeb.cgi, last accessed 19 November 2018) (B). Data show mean values ±SD from three independent biological experiments.

To identify *in planta* sites of IQD5 function, we determined spatio-temporal expression domains of *IQD5* in transgenic *pIQD5*_*short*_*::GFP-GUS* and *pIQD5*_*long*_*::GFP-GUS* reporter lines, in which a 1207 bp and 2201 bp DNA fragment upstream of the translational start site of the *IQD5* gene were fused to the reporter, respectively. Histochemical GUS analysis of *pIQD5*_*short*_*::GFP-GUS* lines throughout development revealed strong promoter activity in cotyledons and leaves, in the vasculature of leaves and the hypocotyl, as well as in the shoot apical meristem (Fig. 1A). In roots, GUS staining was detectable mostly in older parts of the root. In root tips, *IQD5* promoter activity was restricted to the lateral root cap of primary and lateral root meristems. GUS activity was largely absent from reproductive organs, such as flower buds, flowers, siliques, and seeds, and during embryo development. The GUS patterns are consistent with developmental *IQD5* expression data obtained from publicly available microarray data sets (Fig. 1B) (Winter *et al.*, 2007), which confirm higher *IQD5* expression levels in vegetative tissues when compared with reproductive tissues. Similar expression patterns were observed in *pIQD5*_*long*_*::GFP-GUS* lines (Supplementary Fig. S1 available at *JXB* online), suggesting that the 1207 bp fragment was sufficient to report authentic *IQD5* expression patterns. Our analysis thus reveals preferential expression of *IQD5* in vegetative tissues of shoots and roots.

### IQD5–GFP localizes to cortical microtubules

**Fig. 2.**
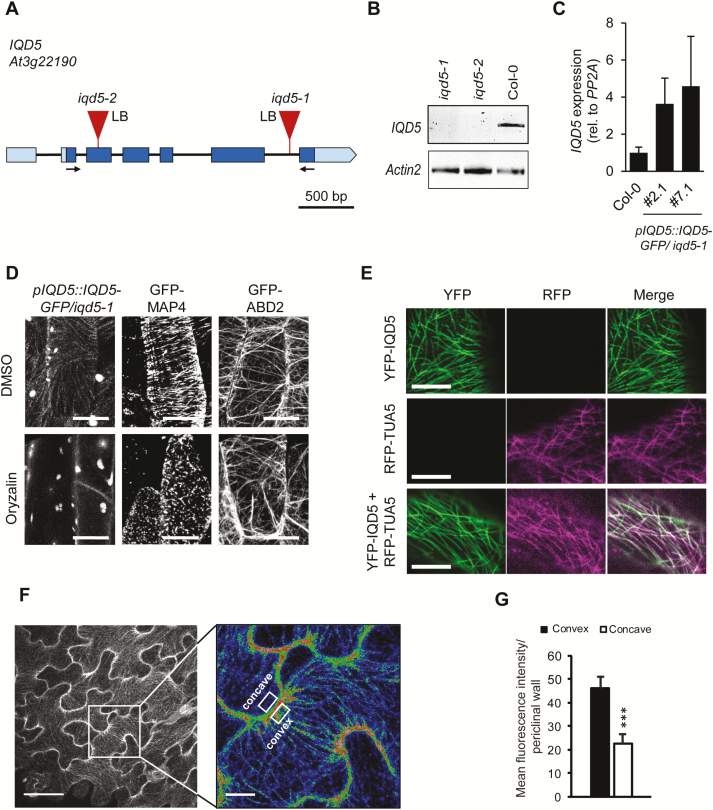
Subcellular localization of IQD5–GFP in transgenic Arabidopsis *pIQD5::IQD5-GFP/iqd5-1* lines. Gene model and position of T-DNA insertions in two independent mutant lines, *iqd5-1* and *iqd5-2* (A). Boxes indicate the 5'UTR and 3'UTR (light blue), and exons (dark blue). Introns are represented by the black line. Loss of *IQD5* full-length transcript in *iqd5-1* and *iqd5-2* plants compared with the WT (Col-0) was validated by RT–PCR (B). Arrows in (A) indicate the position of primers used for amplification of *IQD5* transcripts. *Actin2* was included as a control for cDNA integrity. Relative *IQD5* expression levels were analyzed by qRT-PCR in two independent transgenic *pIQD5::IQD5-GFP/iqd5-1* lines (#2.1 and #7.1) compared with the WT (C). Data show mean values ±SD of three independent biological experiments. Subcellular localization of IQD5–GFP in hypocotyls of transgenic *pIQD5::IQD5-GFP/iqd5-1* seedlings after mock (DMSO) or oryzalin (10 µM) treatment; scale bars=20 µm (D). Subcellular localization of YFP–IQD5 (top), RFP–TUA5 (middle), and of YFP–IQD5 and RFP–TUA5 (bottom) in transient (co-)expression assays in leaves of *N. benthamiana* (E). Transgenic *pCaMV 35S::GFP-MAP4* and *pCaMV 35S::GFP-ABD2* seedlings were included as controls for the microtubule and actin cytoskeleton, respectively. *Z*-stack images of GFP fluorescence in epidermis pavement cells of cotyledons from 5-day-old *pIQD5::IQD5-GFP/iqd5-1* seedlings (F). Overview images (left column) and close-up of lobe regions (right column; fluorescence intensities are shown by false coloring, red, high fluorescence intensity; blue, low fluorescence intensity). Scale bars=50 µm and 5 µm, respectively. Mean fluorescence intensities measured at the convex and concave side of lobe regions in the upper periclinal wall (G). Data show mean values ±SD from a total of 50 lobes, quantified in 10 cells from five seedlings.

To examine the subcellular localization of IQD5, we generated a fluorescent protein fusion construct, in which GFP was fused to the C-terminus of IQD5 within a genomic fragment containing the native *IQD5*_*short*_ promoter (Fig. 2). The *pIQD5::IQD5-GFP* construct was introduced into an *iqd5* knockout background to avoid dosage effects of *IQD5* copy number (Fig. 2A, B). We obtained two independent Arabidopsis T-DNA insertion lines for *IQD5*, which we termed *iqd5-1* and *iqd5-2* (Fig. 2A). RT–PCR analysis revealed the complete absence of full-length *IQD5* transcripts in *iqd5-1* and *iqd5-2* lines when compared with the WT, demonstrating that both T-DNA insertion lines are null mutant alleles (Fig. 2B). Based on macroscopic examination, both *iqd5* mutants were phenotypically indistinguishable from WT plants, as shown for root length and shoot growth (Supplementary Fig. S2). The *iqd5-1* mutant was transformed with the *pIQD5::IQD5-GFP* construct by *Agrobacterium*-mediated floral dip. qRT-PCR analysis of steady-state *IQD5* mRNA levels revealed comparable expression in two independent *pIQD5::IQD5-GFP/iqd5-1* complementation lines, which was moderately higher than in the reference WT (Fig. 2C).

We investigated the subcellular localization of IQD5–GFP by confocal imaging and observed that IQD5 localized in punctate patterns along filamentous structures at the cell cortex of hypocotyl cells, reminiscent of cortical MTs (Fig. 2D). Treatment with oryzalin, a drug that binds to tubulin subunits and prevents MT polymerization (Morejohn *et al.*, 1987), abolished IQD5–GFP localization to filaments, while MTs remained intact upon mock treatment (Fig. 2D). Transgenic *pCaMV 35S::GFP-MAP4* (Marc *et al.*, 1998) and *pCaMV 35S::GFP-ABD2* (Sheahan *et al.*, 2004; Wang *et al.*, 2004) lines were included as controls for the MT and actin cytoskeleton, respectively (Fig. 2D). While oryzalin treatment disrupted MTs decorated with GFP–MAP4, labeled (GFP–ABD2) actin filaments remained intact, demonstrating the efficiency and specificity of the treatment. Co-expression of *pCaMV 35S::YFP-IQD5* with *pCaMV 35S::*RFP-TUA5 in transient expression assays in *N. benthamiana* further corroborated co-localization of YFP–IQD5 with MTs (Fig. 2E). GFP fluorescence was very weak in hypocotyls of *pIQD5::IQD5-GFP/iqd5-1* seedlings. Moderately stronger IQD5–GFP fluorescence was detectable at cortical MT arrays in epidermal PCs of cotyledons (Fig. 2F). PCs adopt highly complex jigsaw puzzle-like shapes with interlocking lobes and necks. Within individual PCs, IQD5–GFP accumulated at the convex side of necks at the interface of anticlinal and outer periclinal walls, as indicated by increased average fluorescence intensities when compared with the concave side (Fig. 2F, G).

### Loss of *IQD5* causes aberrant PC shape

**Fig. 3.**
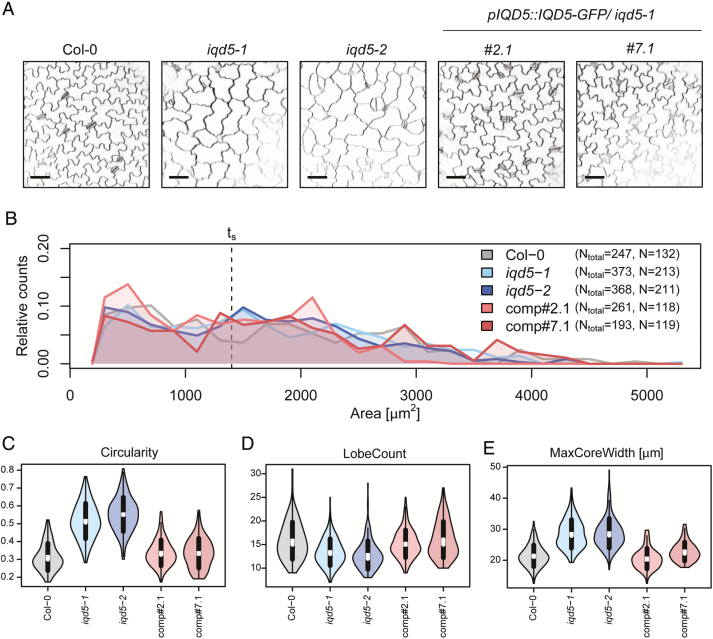
Pavement cell (PC) morphology on the adaxial side of cotyledons from 5-day-old seedlings of the wild type (Col-0), two independent *iqd5* knockout lines (*iqd5-1* and *iqd5-2*), and two independent transgenic complementation lines (*pIQD5:IQD5-GFP/iqd5-1*; lines *#2.1* and *#7.1*). Representative images of PC morphology (A). Cell outlines were visualized with PI. Images are single optical sections. Scale bars=50 µm. Quantification of cell shape features by PaCeQuant (B–H). Relative distribution of cell areas in the analyzed genotypes (B). Numbers in the key refer to the total number of cells from 10 images of the different genotypes. Cells larger than size threshold *t*_s_=1400µm^2^ were used for further analysis. Violin plots of feature distributions for circularity (C), lobe count (D), and maximum core width (MaxCoreWidth, E). Circles and crosses refer to medians and means; the vertical black lines represent the SD (thick lines) and the 95% confidence intervals (thin lines). The width of each violin box represents the local distribution of feature values along the *y*-axis. For an overview of all shape features and statistical analysis, see Supplementary Fig. S3.

To assess whether IQD5 contributes to growth regulation of PCs, we analyzed PC shapes in cotyledons of *iqd5* mutants and of the two *pIQD5::IQD5-GFP/iqd5-1* lines. Cell outlines were visualized by PI staining, and groups of PCs on the adaxial side of cotyledons from 5-day-old seedlings were imaged by confocal microscopy (Fig. 3A). PC shape features were quantified with PaCeQuant (Möller *et al.*, 2017), an ImageJ-based open source tool for fully automatic quantification and graphical visualization of PC shape features. As evidenced by similar areas of individual cells and similar area distributions, expansion was largely unaffected in *iqd5* mutants (Fig. 3B). Cell shapes on the other hand differed strongly in both *iqd5* mutant alleles when compared with the WT or the two independent *pIQD5::IQD5-GFP/iqd5-1* lines. Mutants displayed an increased cellular circularity (Fig. 3C). Circularity values range between 0 and 1, where a circularity value of 1 represents a perfect circle. Increased circularity thus indicates reduced cellular complexity in *iqd5* mutants. Reduced cellular complexity correlates with a moderately reduced average number of lobes from 15 lobes per cell in WT to 13 lobes per cell in *iqd5* mutants (Fig. 3D). In addition, *iqd5* mutants displayed a strongly reduced growth of lobes, indicated by an ~30% reduction of average lobe length (Supplementary Fig. S3A). The width of the cellular core region, measured as the maximum (Fig. 3E) core width, was increased by 22%. Maximum core width provides a clearly defined value as an estimate for the growth restriction of the cellular core region (Möller *et al.*, 2017), which is similar to neck width values manually quantified by, for example, Fu *et al.* (2005). An increased maximum core width indicates reduced growth restriction at neck regions. The phenotypic differences were highly similar between *iqd5-1* and *iqd5-2* mutant alleles. Expression of *pIQD5::IQD5-GFP* in the *iqd5-1* mutant background restored PC shape to WT-like patterns (Fig. 3; Supplementary Fig. S3), which demonstrates functionality of the IQD5–GFP fusion protein and sufficiency of the amplified promoter region for restoring *IQD5* expression levels. Collectively, our data suggest that IQD5 is required for lobe initiation, lobe growth, and growth restriction at neck regions of cotyledon PCs, which is consistent with its predominant localization to cortical MT arrays at necks.

### Cell shape defects in *iqd5* mutants occur early during cotyledon development

**Fig. 4.**
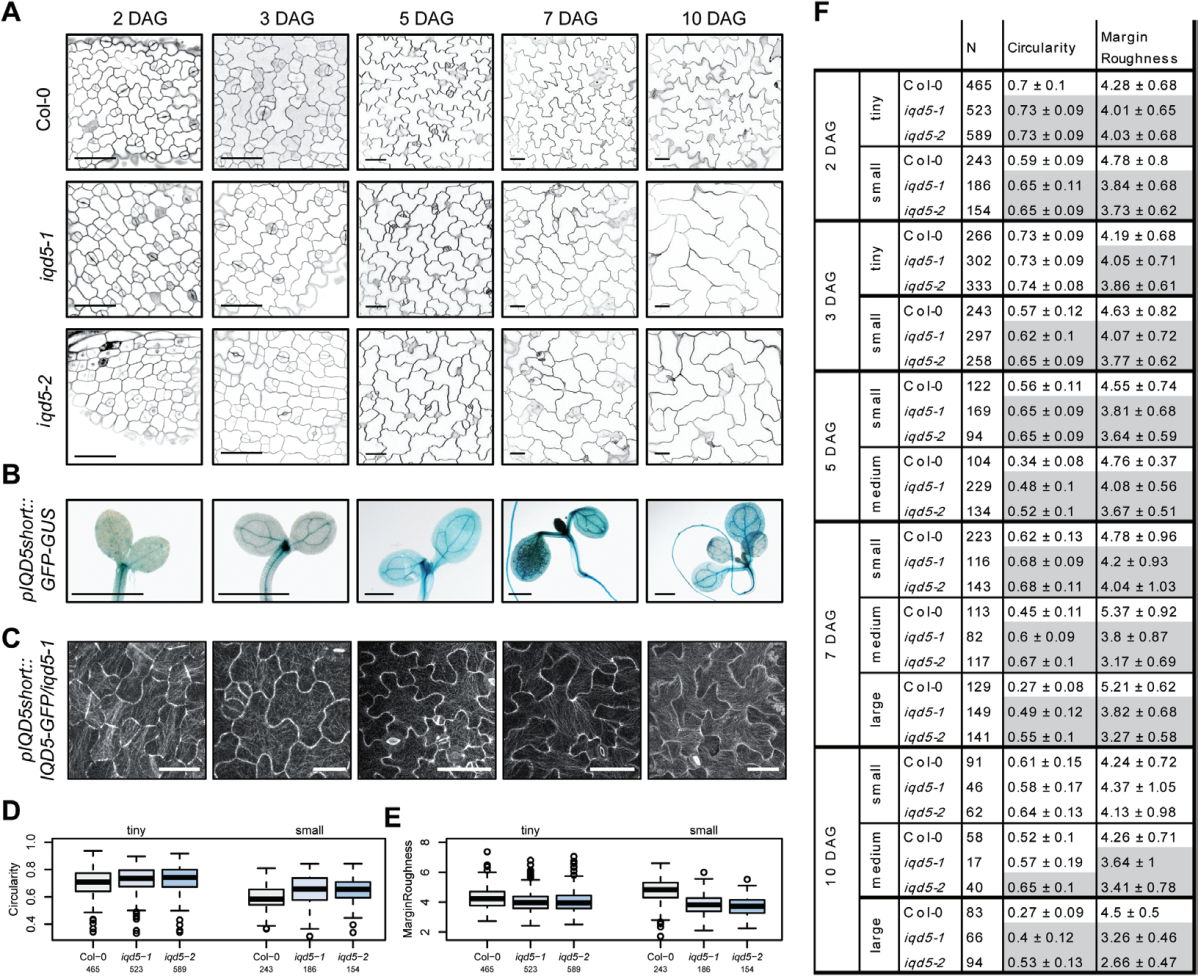
PC morphology during cotyledon development. Representative images of epidermis cells on the adaxial side of cotyledons of WT, *iqd5-1*, and *iqd5-2* seedlings at 2, 3, 5, 7, and 10 days after germination (DAG) (A). Cell outlines were visualized by PI; scale bars=50 µm. Histochemical GUS staining of *pIQD5*_*short*_*::GFP-GUS* seedlings at the indicated time points; scale bars=1 mm (B). Analysis of IQD5–GFP subcellular localization in *pIQD5*_*short*_*::IQD5-GFP/iqd5-1* lines at the indicated time points. Scale bars=20 µm (2 and 3 DAG) and 50 µm (5–10 DAG) (C). Quantification of PC shape parameters by PaCeQuant. Cell populations were grouped according to their size as tiny, 75–240 µm^2^, small, 240–1400 µm^2^, medium 1400–4042 µm^2^, and large ≥4042 µm^2^. Boxplots show feature distributions for circularity (D) and margin roughness (E) in seedlings at 2 DAG. Results are medians; boxes range from the first to third quartile. Feature values for circularity and margin roughness in tiny, small, medium, and large-sized cell populations during cotyledon development (F). Results show mean values ±SD. Statistically significant differences (*P*≤0.05) of *iqd5* mutants relative to the WT are highlighted in gray. For an overview of all shape features and statistical analysis, see Supplementary Fig. S4–S8.

Morphogenesis of PCs in cotyledons is established during distinct phases (Fu *et al.*, 2002; Zhang *et al.*, 2011). In the early phase, 1–3 DAG, lobe formation is initiated and cells start to expand anisotropically, which is followed (3–7 DAG) by diffuse growth and expansion of shape patterns. Growth ceases at later stages (10–18 DAG), and PCs as well as cotyledons reach their final size (Belteton *et al.*, 2018). To determine at which stage IQD5 functions, we studied PC shape in *iqd5* mutants during cotyledon development and imaged WT and *iqd5* mutant seedlings at 2, 3, 5, 7, and 10 DAG (Fig. 4A). During development, the average cell size increased (Supplementary Figs S4–S8), which is consistent with earlier reports (Zhang *et al.*, 2011; Möller *et al.*, 2017). To examine shape and geometries in cell populations of similar sizes, referred to as small, medium, and large, we applied the size thresholds of *t*_s_=1400 µm^2^ and *t*_m_ of 4040 µm^2^ at 3, 5, 7, and 10 DAG. These thresholds were experimentally determined in our previous work (Möller *et al.*, 2017), and resemble cells at early (small), intermediate (medium), and late stages of cellular expansion. To distinguish between very small (tiny) and small cell populations in cotyledons at 2 and 3 DAG, a time span during which lobe formation and anisotropic expansion are initiated, we included an additional size threshold of *t*_tiny_=240 µm^2^. Quantification of PC shape features revealed first differences in cell shapes of *iqd5* mutants already at 2 and 3 DAG (Fig. 4D, E). When compared with the WT, cellular circularity was moderately but significantly increased in both *iqd5* mutant alleles in tiny and in small-sized cell populations (Fig. 4D), and margin roughness, a measure for the (ir-)regularity of local curvature values along the cell contour, as well as average basal lobe length were reduced (Fig. 4E; Supplementary Fig. S4). Similar results were observed in seedlings at 3 DAG (Supplementary Fig. S5). In medium to large-sized cell populations, analyzed in cotyledons between 5 and 10 DAG, phenotypic differences became more pronounced with increasing cell size (Fig. 4F; Supplementary Figs S6–S8). The time series analysis thus suggests important roles for IQD5 already during early phases of PC morphogenesis in cotyledons. Analysis of *pIQD5*_*short*_*::GFP-GUS* and *pIQD5::IQD5-GFP/iqd5-1* lines revealed promoter activity and accumulation of IQD5–GFP at neck regions, respectively, in cotyledons and in the shoot apical meristem between 2 and 10 DAG (Fig. 4B, C). Thus, our data demonstrate that *IQD5* is expressed early during cotyledon development and that loss of *IQD5* causes reduced lobe initiation and anisotropic expansion during early growth phases.

### IQD5 regulates PC shape during embryogenesis and post-embryonic growth

**Fig. 5.**
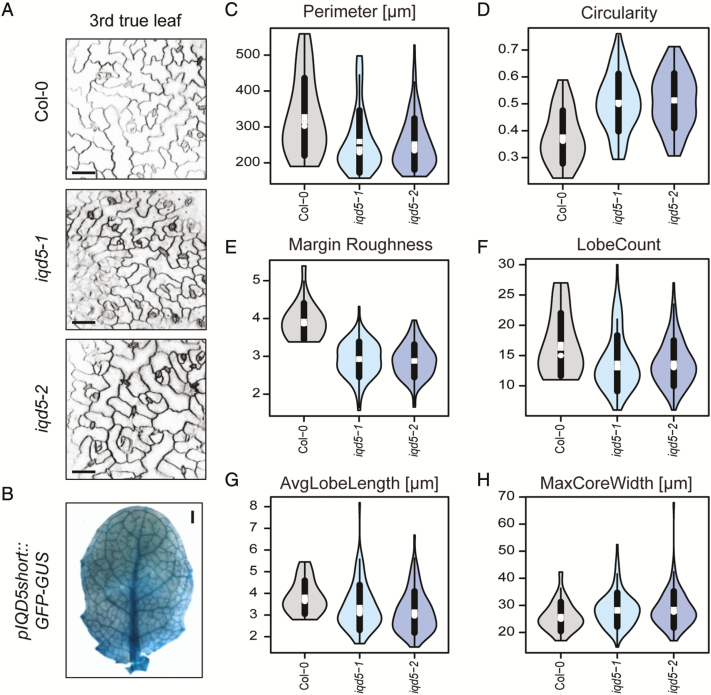
PC shapes in the epidermis of rosette leaves of the WT and *iqd5* mutants. Images are single optical sections of PI-labeled epidermis cells on the adaxial side of the third rosette leaf in 3-week-old plants; scale bars=50 µm (A). Whole-mount GUS staining of the third rosette leaf in 3-week-old *pIQD5*_*short*_*::GFP-GUS* plants; scale bar=1 mm (B). Quantification of PC shape features. Violin plots show feature distributions from *n*=23–110 cells from 9–13 images for perimeter (C), circularity (D), margin roughness (E), lobe count (F), average lobe length (G), and maximum core width (H). Circles and crosses refer to medians and means; the vertical black lines represent the SD (thick lines) and the 95% confidence intervals (thin lines). The width of each violin box represents the local distribution of feature values along the *y*-axis. For an overview of all shape features and statistical analysis, see Supplementary Fig. S9.

Cotyledons resemble true leaves in many aspects, and thus provide a convenient system to study leaf development (Tsukaya *et al.*, 1994). However, while cotyledons emerge in embryogenesis, true leaves post-embryonically differentiate from the shoot apical meristem and, unlike cotyledons, differ in their final leaf shape (Tsukaya, 2002). Moreover, some mutations affect exclusively the development of cotyledons or true leaves (Tsukaya, 1995). To test if *IQD5* also functions in true leaf development, we analyzed PC shape in rosette leaves of 3-week-old plants (Fig. 5A). Morphologically, the first two true leaves in Arabidopsis are similar to cotyledons (Poethig, 1997; Kerstetter and Poethig, 1998), and phenotypes in some rosette leaf-specific mutants are only visible beyond the second true leaf (Guo *et al.*, 2015). To reflect characteristics of true leaves, we thus focused on the third rosette leaf and analyzed PC shape on the adaxial side (Fig. 5C). Quantification of PC shape features revealed similar shape defects in rosette leaves to those observed for cotyledons. Loss of *IQD5* caused a reduced initiation of lobes, as indicated by reduced lobe counts in *iqd5-1* and *iqd5-2* mutants when compared with the WT (Fig. 5F), and the average lobe length of *iqd5* mutants was reduced (Fig. 5G). Reduced formation and growth of lobes were additionally reflected by increased circularity values (Fig. 5D), as well as reduced margin roughness (Fig. 5E) in PCs of *iqd5* mutant plants. Values of minimum (Supplementary Fig. S9) and maximum core width (Fig. 5H) increased, indicative of reduced growth restriction at neck regions. IQD5 thus controls lobe growth and anisotropic expansion in cotyledons and true leaves. In agreement with functions of IQD5 in true leaves, histochemical GUS activity was detectable in *pIQD5*_*short*_*::GFP-GUS* lines within the entire leaf, indicating that *IQD5* is expressed throughout PC growth (Fig. 5B). Taken together, our data identify IQD5 as a novel regulator of leaf epidermis PC shape, which controls growth restriction at necks in embryonic and post-embryonic tissues.

### Reduced growth restriction correlates with altered cellulose deposition

**Fig. 6.**
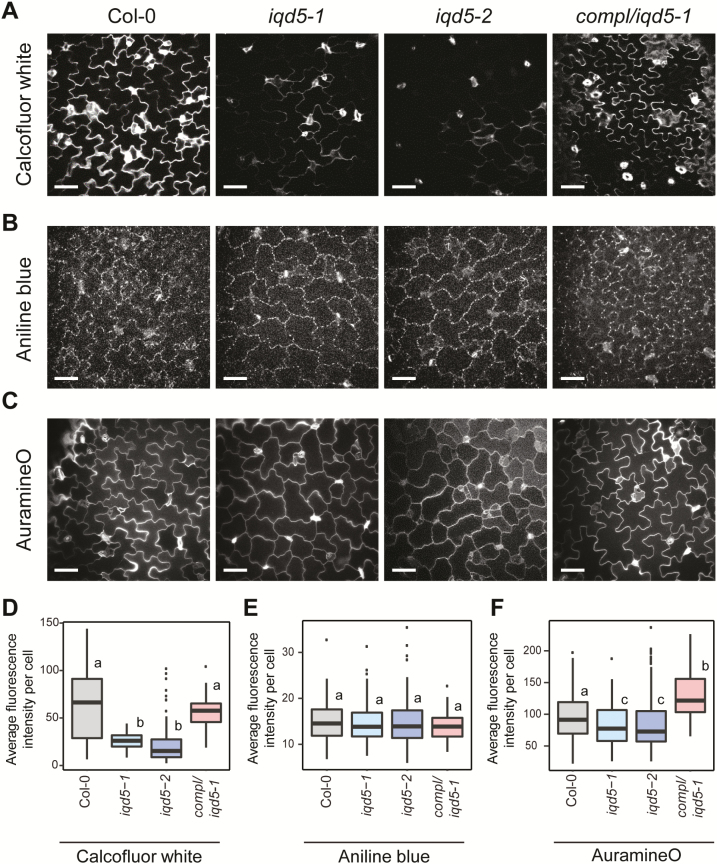
Histochemical analysis of cell wall composition in the WT, the two *iqd5* mutant alleles *iqd5-1* and *iqd5-2*, and in one transgenic *pIQD5::IQD5-GFP/iqd5-1* line. Confocal images show single optical sections of epidermis pavement cells in cotyledons of 5-day-old seedlings. Cellulose staining by calcofluor white (A), aniline blue staining of callose (B), and auramine O staining of the cuticle (C). Scale bars=50 µm. Quantification of relative fluorescence intensities along the anticlinal cell wall (D–F). Shown are medians; boxes range from the first to third quartile. Different letters denote a statistically significant difference by one-way ANOVA, *P*<0.01.

MTs guide CSCs and determine the deposition and direction of newly forming cellulose fibrils in the cell wall (Paredez *et al.*, 2006; Gutierrez *et al.*, 2009; Endler and Persson, 2011). During cell expansion, cellulose fibrils are aligned perpendicular to the growth axis and promote anisotropic expansion. Because IQD5–GFP labels MTs, and mutants defective in *iqd5* display shape defects reminiscent of decreased growth restriction at necks, we aimed to investigate whether IQD5 affects cellulose deposition. Staining with calcofluor white, a dye used for visualization of cellulose fibrils (Seagull, 1986; Anderson *et al.*, 2010), revealed reduced staining intensities in *iqd5-1* and *iqd5-2* when compared with the WT, which were reverted to WT levels in the complementation line (Fig. 6A). To assess differences in fluorescence intensities at anticlinal cell walls quantitatively, we segmented the contour of individual cells after visualization of cell walls by co-staining with PI using the segmentation mode implemented in PaCeQuant. We measured an ~45% reduction of calcofluor white fluorescence intensities along the cell contour of *iqd5* mutant cells compared with the WT and the complementation line (Fig. 6D). Reduced intensities suggest reduced deposition of cellulose in anticlinal cell walls of PCs. Calcofluor white, however, does not discriminate between β-1,3- and β-1,4-glucan chains (Anderson *et al.*, 2010), which are the building blocks of callose and cellulose, respectively. To test whether loss of *IQD5* specifically affects cellulose deposition, we included aniline blue staining to visualize callose (Wood, 1984) and quantified fluorescence intensities. No differences in callose deposition were observed in the mutants when compared with the WT or the complementation lines (Fig. 6B, E). Similarly, only minor differences (5–10%) in fluorescence intensities were observed upon auramine O staining, which labels the cuticle (Considine and Knox, 1979) (Fig. 6C, F). Taken together, our data suggest that the reduced calcofluor white signals are not an artifact of reduced penetration or uptake of the dyes due to general defects in cell wall composition, and probably reflect reduced cellulose deposition caused by the loss of *IQD5*.

### IQD5-dependent recruitment of CaM to cortical microtubules

**Fig. 7.**
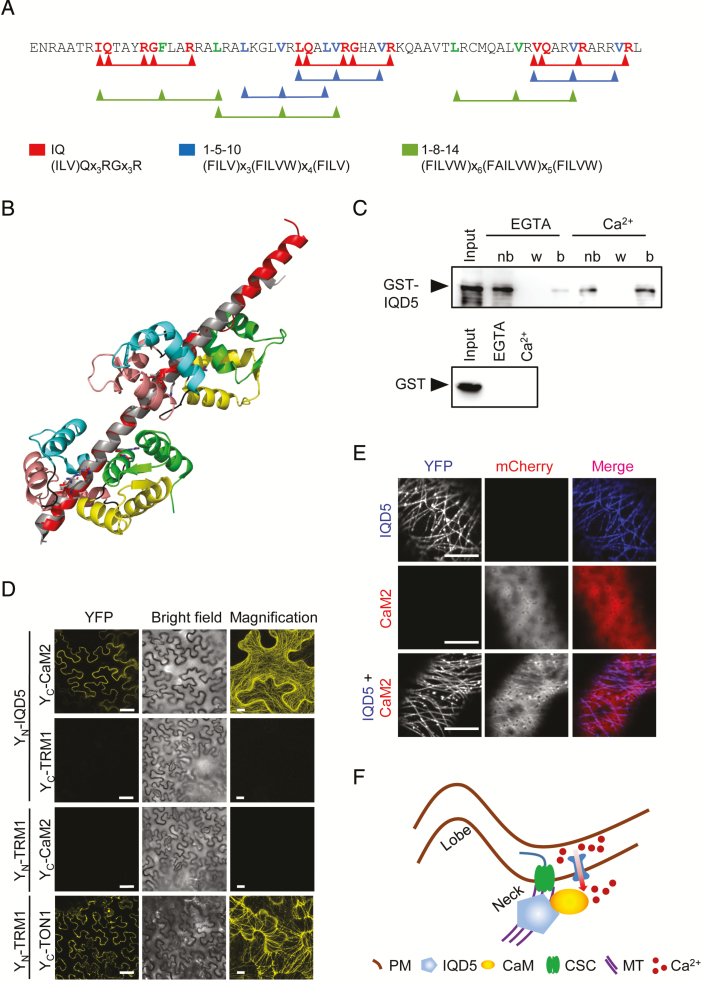
Calmodulin (CaM) binding properties of IQD5. Amino acid sequence of the IQ67 domain of IQD5 (A). IQ motifs implicated in apoCaM (IQ) and Ca^2+^–CaM (1-5-10 and 1-8-14) binding are highlighted in red, blue, and green, respectively. Structural alignment of the IQ67 domain of IQD5 (red) and the IQ motif-containing domain of myosin (gray), together with two apo-CaM proteins (first, second, third, and fourth EF hand in green, yellow, salmon, and cyan, respectively (aligned and fitted with PyMol) (B). *In vitro* pull-down of recombinant GST–IQD5 and as control of GST alone expressed in *E. coli* with bovine CaM immobilized on agarose beads in the presence (Ca^2+^) and absence (EGTA) of calcium; nb, not bound; w, last wash; b, bead-immobilized fraction (C). *In planta* interaction of IQD5 with CaM (D, E). BiFC assays between Y_N_–IQD5 and Y_C_–CaM2 in leaves of *N. benthamiana* (D). Combinations of Y_N_–IQD5 with Y_C_–TRM1 and Y_C_–CaM2 with Y_N_–TRM1 served as negative controls. Y_N_–TRM1 and Y_C_–TON1 were included as positive control. Single optical sections of YFP fluorescence (left column) and corresponding bright field images (center column). Scale bars=50 µm. Right column, close-up *Z*-stack images of YFP fluorescence; scale bars=10 µm. Subcellular localization of mCherry–CaM2 (top), YFP–IQD5 (middle), and of YFP–IQD5 and mCherry–CaM2 (bottom) in transient (co-)expression assays in leaves of *N. benthamiana* (E). Scale bars=5 µm. Proposed model of IQD5 function in pavement cell morphogenesis (F). IQD5 localizes to microtubules and is required for growth restriction at neck regions, possibly by affecting cellulose deposition along anticlinal cell walls. Interaction of IQD5 with CaM at microtubules points to important roles for Ca^2+^ signaling during shape establishment.

A hallmark of IQD proteins is the presence of their eponymous IQ67 domain, which contains a repetitive arrangement of predicted CaM-binding motifs (Abel *et al.*, 2005). In IQD5, the IQ67 domain contains three copies each of three different classes of CaM-interacting motifs, including the IQ motif and motifs of the 1-5-10 and 1-8-14 classes, with presumed roles for binding to apo-CaM (IQ) and holo-CaM (1-5-10 and 1-8-14), respectively (Fig. 7A). Homology modeling of IQD5 indicates that the IQ67 domain adopts an α-helical fold (Fig. 7B), similar to the apo-CaM-binding domain of myosin (Houdusse *et al.*, 2006), and potentially interacts simultaneously with more than one CaM polypeptide. To assess whether IQD5 is a functional CaM target, we performed *in vitro* CaM binding assays (Fig. 7C). We expressed GST-tagged IQD5 and the GST core as a control in *E. coli* to investigate interaction with immobilized bovine CaM in the presence (Ca^2+^) and absence (EGTA) of calcium. GST–IQD5, but not GST, co-sedimented with apo-CaM, and CaM binding of GST–IQD5 was enhanced in the presence of Ca^2+^. CaM binding thus is independent of the GST tag, and the predicted CaM-binding motifs (Fig. 7A) are functional in mediating interaction with both states of CaM, the Ca^2+^-free apo-CaM and Ca^2+^-bound holo-CaM (Fig. 7C). To gain insight into subcellular sites of IQD5 interaction with CaM, we performed BiFC analyses. N-terminal fusions of IQD5 to the N-terminal half of YFP (Y_N_–IQD5) were transiently co-expressed with N-terminal fusions of CaM2 to the C-terminal half of YFP (Y_C_–CaM2) in *N. benthamiana* leaves by infiltration with *Agrobacterium* harboring the respective plasmids. As controls, we included Y_N_ and Y_C_ fusions of TON1 RECRUITMENT MOTIF1 (TRM1), a member of a plant-specific class of MAPs that interacts with TONNEAU1 (TON1) *in planta* (Drevensek *et al.*, 2012). Recovery of YFP fluorescence was visible along the MT lattice between Y_N_–IQD5 and Y_C_–CaM2, and between Y_N_–TRM1 and Y_C_–TON1, which served as positive control (Fig. 7D). No fluorescence complementation was detectable in the negative controls, in which Y_N_–IQD5 and Y_C_–CaM2 were combined with Y_C_–TRM1 and Y_N_–TRM1, respectively, demonstrating specificity of the BiFC assay. Additionally, CaM binding at MTs was validated in co-expression assays (Fig. 7E). Expression of *pCaMV 35S::mCherry-CaM2* resulted in cytosolic accumulation of mCherry–CaM2, consistent with previous reports (Bürstenbinder *et al.*, 2013). Upon co-expression with YFP–IQD5, mCherry–CaM2 re-localized to cortical MTs (Fig. 7E). Important roles for Ca^2+^ in regulation of PC shape are supported by altered PC morphology in seedlings grown under elevated concentrations of Ca^2+^ (Supplementary Fig. S10). High external Ca^2+^ resulted in increased circularity and an increased minimum and maximum core width, indicative of a reduced growth restriction at neck regions. Thus, our data point to roles of IQD5 in CaM recruitment to cortical MTs, and provide the first indications for CaM-dependent Ca^2+^ signaling in shape development of leaf epidermis PCs (Fig. 7F).

## Discussion

Plant-specific IQD families emerged as one of the largest class of CaM targets with proposed roles in linking Ca^2+^ signaling to the regulation of plant growth and development (Bürstenbinder *et al.*, 2017*b*). Despite growing evidence for important roles as cellular scaffolds at the MT cytoskeleton, the precise molecular mechanisms of IQD functions are still enigmatic (Bürstenbinder *et al.*, 2017*a*). Here, we provide experimental evidence that (i) positions IQD5 at cortical MT arrays in vegetative tissues (Figs 1, 2) and (ii) identifies IQD5 as a novel regulator of shape establishment in epidermal PCs of cotyledons and leaves (Figs 3–5). We further show that (iii) phenotypes in *iqd5* mutants correlate with alteration of cell wall properties (Fig. 6) and (iv) provide experimental evidence for interaction of IQD5 with CaM2 *in vitro* and *in planta* (Fig. 7). Thus, our data support roles for IQD5 in regulation of MTs and cellulose deposition during PC morphogenesis. We further provide the first evidence for roles of Ca^2+^ signaling in the spatial co-ordination of cell expansion during interdigitated growth of PCs.

PCs are the most abundant cell type in the leaf epidermis, which are characterized by their jigsaw puzzle-like shape in Arabidopsis and in several other plant species (Ivakov and Persson, 2013; Jacques *et al.*, 2014). Their morphogenesis relies on lobe initiation and anisotropic expansion to generate the complex multi-lobed shapes of PCs (Zhang *et al.*, 2011). Our study establishes IQD5 as a novel regulator of PC shape formation as revealed by reduced lobe growth and growth restriction of neck regions in PCs of *iqd5* mutants. Lobe initiation, however, is only slightly reduced, and cell size as well as overall growth are unaffected, which indicates that IQD5 specifically functions in control of anisotropic expansion of PCs. Consistent with roles during anisotropic expansion, shape defects in *iqd5* mutants are established during early growth phases (i.e. in cotyledons at 2 DAG) at which lobe formation and lobe growth are initiated, and persist during later growth phases, in which cells expand within the lateral cell borders defined during the early growth phase (Zhang *et al.*, 2011). A role for IQD5 in regulating anisotropic expansion is further supported by its expression pattern identified in *pIQD5::GFP-GUS* reporter lines, which revealed uniform promoter activity within cotyledons and leaves. Similarly, growth and shape changes of PCs occur throughout the entire leaf, and growth rates display large heterogeneity between neighboring cells within expanding leaves (Elsner *et al.*, 2012). In contrast, cell cycle activity ceases in a longitudinal gradient during leaf maturation (Asl *et al.*, 2011). At later growth stages, cell division is restricted to the basal part of cotyledons and leaves, as shown by analysis of the cell division marker CYCLINB1;1 (CYCB1;1) in transgenic *pCYC1;1::GUS* reporter lines (Ferreira *et al.*, 1994; Dhondt *et al.*, 2010; Carter *et al.*, 2017). The combined analysis of mutant phenotypes and of spatio-temporal expression domains thus establishes IQD5 as a novel factor controlling anisotropic growth of leaf epidermis PCs.

The plant MT cytoskeleton plays important roles in regulation of anisotropic expansion during PC morphogenesis (Fu *et al.*, 2002, 2005; Jacques *et al.*, 2014). Disturbances in MT organization, stability, or dynamics by pharmacological agents or by mutations in MAPs, such as TRM2/LONGIFOLIA1 or KATANIN (KTN1), reduce cellular complexity of PC morphogenesis (Lee *et al.*, 2006; Lin *et al.*, 2013; Akita *et al.*, 2015). Potential roles of IQD proteins as MAPs that control cell expansion are supported by our previous work, which revealed altered MT organization and PC shape in transgenic plants ectopically overexpressing *IQD11*, *IQD14*, or *IQD16* (Bürstenbinder *et al.*, 2017*b*). Moreover, altered expression of *IQD* genes is linked to regulation of grain size in rice (Duan *et al.*, 2017; Liu *et al.*, 2017; Yang *et al.*, 2018, Preprint) and fruit shape in tomato (Xiao *et al.*, 2008), water melon (*Citrullus lanatus* L.) (Dou *et al.*, 2018), and cucumber (*Cucumis sativus* L.) (Pan *et al*., 2018), suggesting that IQDs are key determinants of cell and organ shape (Bürstenbinder *et al.*, 2017*a*). Here, by analysis of GFP fluorescence in transgenic *pIQD5::IQD5-GFP/iqd5-1* lines, we demonstrate subcellular localization of IQD5–GFP to cortical MT arrays in leaf epidermal PCs. Functionality of the GFP-tagged IQD5 protein is indicated by efficient complementation of PC shape defects in *pIQD5::IQD5-GFP/iqd5-1* lines. MT localization was validated by oryzalin treatment in transgenic Arabidopsis plants and by co-localization of YFP–IQD5 with the MT marker RFP–TUA5 in transient expression assays in *N. benthamiana*. Our work thus for the first time identifies a direct link between MTs and cell expansion in an *iqd* knockout mutant. Notably, in contrast to most reported mutants with defects in PC shape that have pleiotropic effects, including reduced plant growth, organ twisting, or swelling of cells (Qian *et al.*, 2009), *iqd5* mutants are macroscopically indistinguishable from the WT. Specific defects in PC morphogenesis thus indicate limited functional redundancy and compensation between the 33 IQD family members in Arabidopsis and point to unique roles for IQD5 in PC morphogenesis.

Quantification of fluorescence intensities along the outer periclinal cell wall suggests accumulation of IQD5–GFP at convex sides of indenting neck regions. Our findings are consistent with *iqd5* mutant phenotypes and patterns of subcellular IQD5 localization reported in an independent study, which was published while our work was under revision (Liang *et al.*, 2018). Similar to IQD5–GFP, preferential accumulation of MT bundles at neck regions has been reported in several studies, and MT accumulation correlates with reduced growth in the neck regions (Fu *et al.*, 2005; Sampathkumar *et al.*, 2014; Armour *et al.*, 2015). Cortical MTs serve as tracks for PM-localized CSCs and thereby determine the direction of cellulose deposition and consequently cell expansion (Endler and Persson, 2011). Here, using histochemical staining, we show that *iqd5* mutants accumulate reduced amounts of cellulose in their anticlinal walls while callose deposition and cuticle formation are unaffected. Plants with impaired cellulose deposition, for example upon cellulase treatment or in mutants of the cellulose synthase AtCesA1, display strongly reduced lobing of PCs (Higaki *et al.*, 2016; Majda *et al.*, 2017), similar to phenotypes in *iqd5* lines. Our data suggest that IQD5 is required for efficient cellulose deposition, for example by controlling MT dynamics or organization, thereby affecting cellulose synthesis. In agreement with this hypothesis, Liang *et al.* (2018) reported increased disorder of cortical MTs in *iqd5* mutants when compared with the WT and provide first evidence for functions of IQD5 in stabilization of MTs. Alternatively, IQD5 may mediate coupling of cellulose synthase movement to MT tracks, possibly by direct interaction with KINESIN LIGHT CHAIN-RELATED (KLCR)/CELLULOSE MICROTUBULE UNCOUPLING (CMU) family members. KLCRs mediate PM tethering of MTs to stabilize cortical MTs against the pushing forces of CSCs (Liu *et al.*, 2016). Arabidopsis IQD1, IQD2, and IQD23 interact with KLCR family members in yeast, and IQD1 recruits KLCR1 to MTs in transient expression assays in *N. benthamiana* (Mukhtar *et al.*, 2011; Bürstenbinder *et al.*, 2013). Thus, the prospect arises that IQD:KLCR modules collectively co-ordinate MT organization and lateral stability of cortical MTs at the PM–MT nexus.

A hallmark of IQD families is their ability to bind CaM Ca^2+^ sensors, which suggests important roles for IQDs in linking CaM-mediated Ca^2+^ signaling to the regulation of the MT cytoskeleton via as yet unknown mechanisms (Abel *et al.*, 2005, Hepler, 2016; Bürstenbinder *et al.*, 2017*b*). The phenotypes in *iqd5* mutants, together with the IQD5-dependent recruitment of CaM to cortical MTs, provide the first experimental evidence for functions of Ca^2+^ signaling in PC morphogenesis, probably via CaM/CMLs. Roles for Ca^2+^ during PC morphogenesis are further supported by altered PC shapes in response to elevated exogenous Ca^2+^ supply. Interestingly, IQD5 interacts *in vitro* with both states of CaM, the Ca^2+^-free and Ca^2+^-bound apo- and holoCaM, respectively. Similarly, IQD1 and IQD20 interact with apoCaM and holoCaM *in vitro* (Abel *et al.*, 2005; Bürstenbinder *et al.*, 2013), which suggests functionality of the distinct CaM-binding motifs within the IQ67 domain. The repetitive alignment of multiple CaM-binding motifs may facilitate interaction with several CaM/CMLs simultaneously, in which individual CaM-binding motifs differentially contribute to CaM/CML binding. Additionally, CaM/CMLs may exert specific effects on IQD5 depending on their Ca^2+^ occupancy, which adds another potential layer of Ca^2+^-dependent regulation. Identification of *in planta* IQD5-interacting CaM/CMLs, however, will be challenging because large multigene families of 7 and 50 members code for CaMs and CMLs in Arabidopsis, respectively, and many *CaM/CML* genes are expressed in cotyledons and leaves (Supplementary Fig. S11). Ca^2+^ signals are rapidly generated by exogenous application of several phytohormones, including auxin and cytokinin (Saunders and Hepler, 1981; Vanneste and Friml, 2013), which are key regulators of PC morphogenesis that antagonistically activate Rho-like GTPases from plants (ROPs) in lobes and necks, respectively (Fu *et al*., 2002, 2005, 2009). IQD5 and related proteins of the IQD family may constitute promising candidates for integrating upstream signals (e.g. from phytohormones) into the reorganization of MT arrays, possibly via phytohormone-induced Ca^2+^ signals (Bürstenbinder *et al.*, 2017*a*; this study). Lastly, a recent study by Sugiyama *et al.* (2017) provides first indications for functions of IQD13 in spatial control of ROP signaling domains required for cell wall patterning during vessel development. A similar mechanism might apply to IQD5 during PC shape formation, thereby providing a potential link between phytohormone actions, Ca^2+^ signaling, and ROP GTPase activation. Collectively, our work identifies IQD5 as a novel regulator of PC shape and a potential hub for co-ordination of cellular signaling, cytoskeletal reorganization, and cell wall remodeling. Our work thus provides a framework for future mechanistic studies of cellular signaling networks at the cell wall–PM–MT continuum, which will aid a more holistic understanding of cellular processes guiding shape complexity.

## Supplementary data

Supplementary data are available at *JXB* online.

Fig. S1. *IQD5* expression analysis in *pIQD5*_*long*_*::GFP-GUS* reporter lines.

Fig. S2. Macroscopic analysis of growth parameters in the WT and *iqd5* mutants.

Fig. S3. Quantification and statistical analysis of PC shape features in 5-day-old seedlings of the WT and *iqd5* mutants.

Fig. S4. Quantification and statistical analysis of PC shape in cotyledons at 2 DAG.

Fig. S5. Quantification and statistical analysis of PC shape in cotyledons at 3 DAG.

Fig. S6. Quantification and statistical analysis of PC shape in cotyledons at 5 DAG.

Fig. S7. Quantification and statistical analysis of PC shape in cotyledons at 7 DAG.

Fig. S8. Quantification and statistical analysis of PC shape in cotyledons at 10 DAG.

Fig. S9. Quantification and statistical analysis of PC shape in true leaves.

Fig. S10. Calcium-dependent changes in PC shape.

Fig. S11. *In silico* expression analysis of Arabidopsis CaM/CMLs.

## Supplementary Material

Supplementary Material

## References

[CIT0001] Abel S, Savchenko T, Levy M 2005 Genome-wide comparative analysis of the *IQD* gene families in *Arabidopsis thaliana* and *Oryza sativa*. BMC Evolutionary Biology 5, 72. 16368012 10.1186/1471-2148-5-72 PMC1368998

[CIT0002] Akhmanova A, Hammer JA 3rd 2010 Linking molecular motors to membrane cargo. Current Opinion in Cell Biology 22, 479–487. 20466533 10.1016/j.ceb.2010.04.008 PMC3393125

[CIT0003] Akhmanova A, Steinmetz MO 2008 Tracking the ends: a dynamic protein network controls the fate of microtubule tips. Nature Reviews. Molecular Cell Biology 9, 309–322. 18322465 10.1038/nrm2369

[CIT0004] Akita K, Higaki T, Kutsuna N, Hasezawa S 2015 Quantitative analysis of microtubule orientation in interdigitated leaf pavement cells. Plant Signaling & Behavior 10, e1024396. 26039484 10.1080/15592324.2015.1024396 PMC4622981

[CIT0005] Anderson CT, Carroll A, Akhmetova L, Somerville C 2010 Real-time imaging of cellulose reorientation during cell wall expansion in Arabidopsis roots. Plant Physiology 152, 787–796. 19965966 10.1104/pp.109.150128 PMC2815888

[CIT0006] Armour WJ, Barton DA, Law AM, Overall RL 2015 Differential growth in periclinal and anticlinal walls during lobe formation in Arabidopsis cotyledon pavement cells. The Plant Cell 27, 2484–2500. 26296967 10.1105/tpc.114.126664 PMC4815096

[CIT0007] Asl LK, Dhondt S, Boudolf V, Beemster GT, Beeckman T, Inzé D, Govaerts W, De Veylder L 2011 Model-based analysis of Arabidopsis leaf epidermal cells reveals distinct division and expansion patterns for pavement and guard cells. Plant Physiology 156, 2172–2183. 21693673 10.1104/pp.111.181180 PMC3149966

[CIT0008] Bayer EM, Sparkes I, Vanneste S, Rosado A 2017 From shaping organelles to signalling platforms: the emerging functions of plant ER–PM contact sites. Current Opinion in Plant Biology 40, 89–96. 28865976 10.1016/j.pbi.2017.08.006

[CIT0009] Belteton SA, Sawchuk MG, Donohoe BS, Scarpella E, Szymanski DB 2018 Reassessing the roles of PIN proteins and anticlinal microtubules during pavement cell morphogenesis. Plant Physiology 176, 432–449. 29192026 10.1104/pp.17.01554 PMC5761804

[CIT0010] Bringmann M, Li E, Sampathkumar A, Kocabek T, Hauser MT, Persson S 2012 POM-POM2/cellulose synthase interacting1 is essential for the functional association of cellulose synthase and microtubules in Arabidopsis. The Plant Cell 24, 163–177. 22294619 10.1105/tpc.111.093575 PMC3289571

[CIT0011] Bürstenbinder K, Mitra D, Quegwer J 2017 *a* Functions of IQD proteins as hubs in cellular calcium and auxin signaling: a toolbox for shape formation and tissue-specification in plants?Plant Signaling & Behavior 12, e1331198. 28534650 10.1080/15592324.2017.1331198 PMC5566250

[CIT0012] Bürstenbinder K, Möller B, Plötner R, Stamm G, Hause G, Mitra D, Abel S 2017 *b* The IQD family of calmodulin-binding proteins links calcium signaling to microtubules, membrane subdomains, and the nucleus. Plant Physiology 173, 1692–1708. 28115582 10.1104/pp.16.01743 PMC5338658

[CIT0013] Bürstenbinder K, Rzewuski G, Wirtz M, Hell R, Sauter M 2007 The role of methionine recycling for ethylene synthesis in Arabidopsis. The Plant Journal 49, 238–249. 17144895 10.1111/j.1365-313X.2006.02942.x

[CIT0014] Bürstenbinder K, Savchenko T, Müller J, Adamson AW, Stamm G, Kwong R, Zipp BJ, Dinesh DC, Abel S 2013 Arabidopsis calmodulin-binding protein IQ67-domain 1 localizes to microtubules and interacts with kinesin light chain-related protein-1. Journal of Biological Chemistry 288, 1871–1882. 23204523 10.1074/jbc.M112.396200 PMC3548496

[CIT0015] Carter R, Sánchez-Corrales YE, Hartley M, Grieneisen VA, Marée AFM 2017 Pavement cells and the topology puzzle. Development 144, 4386–4397. 29084800 10.1242/dev.157073 PMC5769637

[CIT0016] Chen X, Grandont L, Li H, Hauschild R, Paque S, Abuzeineh A, Rakusová H, Benkova E, Perrot-Rechenmann C, Friml J 2014 Inhibition of cell expansion by rapid ABP1-mediated auxin effect on microtubules. Nature 516, 90–93. 25409144 10.1038/nature13889 PMC4257754

[CIT0017] Clough SJ, Bent AF 1998 Floral dip: a simplified method for *Agrobacterium*-mediated transformation of *Arabidopsis thaliana*. The Plant Journal 16, 735–743. 10069079 10.1046/j.1365-313x.1998.00343.x

[CIT0018] Considine JA, Knox RB 1979 Development and histochemistry of the cells, cell walls, and cuticle of the dermal system of fruit of the grape, *Vitis vinifera* L. Protoplasma 99, 347–365.

[CIT0019] DeLano WL 2009 PyMOL molecular viewer: updates and refinements. Abstracts of Papers of the American Chemical Society 238.

[CIT0020] Dhondt S, Coppens F, De Winter F, Swarup K, Merks RM, Inzé D, Bennett MJ, Beemster GT 2010 SHORT-ROOT and SCARECROW regulate leaf growth in Arabidopsis by stimulating S-phase progression of the cell cycle. Plant Physiology 154, 1183–1195. 20739610 10.1104/pp.110.158857 PMC2971598

[CIT0021] Dou J, Zhao S, Lu X, He N, Zhang L, Ali A, Kuang H, Liu W 2018 Genetic mapping reveals a candidate gene (ClFS1) for fruit shape in watermelon (*Citrullus lanatus* L.). Theoretical and Applied Genetics 131, 947–958. 29362832 10.1007/s00122-018-3050-5

[CIT0022] Drevensek S, Goussot M, Duroc Y, et al 2012 The Arabidopsis TRM1–TON1 interaction reveals a recruitment network common to plant cortical microtubule arrays and eukaryotic centrosomes. The Plant Cell 24, 178–191. 22286137 10.1105/tpc.111.089748 PMC3289559

[CIT0023] Duan P, Xu J, Zeng D, et al 2017 Natural variation in the promoter of *GSE5* contributes to grain size diversity in rice. Molecular Plant 10, 685–694. 28366824 10.1016/j.molp.2017.03.009

[CIT0024] Elsner J, Michalski M, Kwiatkowska D 2012 Spatiotemporal variation of leaf epidermal cell growth: a quantitative analysis of *Arabidopsis thaliana* wild-type and triple cyclinD3 mutant plants. Annals of Botany 109, 897–910. 22307569 10.1093/aob/mcs005 PMC3310487

[CIT0025] Endler A, Persson S 2011 Cellulose synthases and synthesis in Arabidopsis. Molecular Plant 4, 199–211. 21307367 10.1093/mp/ssq079

[CIT0026] Feng L, Chen Z, Ma H, Chen X, Li Y, Wang Y, Xiang Y 2014 The *IQD* gene family in soybean: structure, phylogeny, evolution and expression. PLoS One 9, e110896. 25343341 10.1371/journal.pone.0110896 PMC4208818

[CIT0027] Ferreira PC, Hemerly AS, Engler JD, van Montagu M, Engler G, Inzé D 1994 Developmental expression of the arabidopsis cyclin gene cyc1At. The Plant Cell 6, 1763–1774. 7866022 10.1105/tpc.6.12.1763 PMC160560

[CIT0028] Fu Y, Gu Y, Zheng Z, Wasteneys G, Yang Z 2005 Arabidopsis interdigitating cell growth requires two antagonistic pathways with opposing action on cell morphogenesis. Cell 120, 687–700. 15766531 10.1016/j.cell.2004.12.026

[CIT0029] Fu Y, Li H, Yang Z 2002 The ROP2 GTPase controls the formation of cortical fine F-actin and the early phase of directional cell expansion during Arabidopsis organogenesis. The Plant Cell 14, 777–794. 11971134 10.1105/tpc.001537 PMC150681

[CIT0030] Fu Y, Xu T, Zhu L, Wen M, Yang Z 2009 A ROP GTPase signaling pathway controls cortical microtubule ordering and cell expansion in Arabidopsis. Current Biology 19, 1827–1832. 19818614 10.1016/j.cub.2009.08.052 PMC2933814

[CIT0031] Gantner J, Ordon J, Ilse T, Kretschmer C, Gruetzner R, Löfke C, Dagdas Y, Bürstenbinder K, Marillonnet S, Stuttmann J 2018 Peripheral infrastructure vectors and an extended set of plant parts for the Modular Cloning system. PLoS One 13, e0197185. 29847550 10.1371/journal.pone.0197185 PMC5976141

[CIT0032] Gehl C, Waadt R, Kudla J, Mendel RR, Hänsch R 2009 New GATEWAY vectors for high throughput analyses of protein–protein interactions by bimolecular fluorescence complementation. Molecular Plant 2, 1051–1058. 19825679 10.1093/mp/ssp040

[CIT0033] Guo X, Qin Q, Yan J, Niu Y, Huang B, Guan L, Li Y, Ren D, Li J, Hou S 2015 TYPE-ONE PROTEIN PHOSPHATASE4 regulates pavement cell interdigitation by modulating PIN-FORMED1 polarity and trafficking in Arabidopsis. Plant Physiology 167, 1058–1075. 25560878 10.1104/pp.114.249904 PMC4348754

[CIT0034] Gutierrez R, Lindeboom JJ, Paredez AR, Emons AM, Ehrhardt DW 2009 Arabidopsis cortical microtubules position cellulose synthase delivery to the plasma membrane and interact with cellulose synthase trafficking compartments. Nature Cell Biology 11, 797–806. 19525940 10.1038/ncb1886

[CIT0035] Hardham AR, Takemoto D, White RG 2008 Rapid and dynamic subcellular reorganization following mechanical stimulation of Arabidopsis epidermal cells mimics responses to fungal and oomycete attack. BMC Plant Biology 8, 63. 18513448 10.1186/1471-2229-8-63 PMC2435237

[CIT0036] Hepler PK 2005 Calcium: a central regulator of plant growth and development. The Plant Cell 17, 2142–2155. 16061961 10.1105/tpc.105.032508 PMC1182479

[CIT0037] Hepler PK 2016 The cytoskeleton and its regulation by calcium and protons. Plant Physiology 170, 3–22. 26722019 10.1104/pp.15.01506 PMC4704593

[CIT0038] Higaki T, Kutsuna N, Akita K, Takigawa-Imamura H, Yoshimura K, Miura T 2016 A theoretical model of jigsaw-puzzle pattern formation by plant leaf epidermal cells. PLoS Computational Biology 12, e1004833. 27054467 10.1371/journal.pcbi.1004833 PMC4824374

[CIT0039] Horio T, Murata T 2014 The role of dynamic instability in microtubule organization. Frontiers in Plant Science 5, 511. 25339962 10.3389/fpls.2014.00511 PMC4188131

[CIT0040] Houdusse A, Gaucher JF, Krementsova E, Mui S, Trybus KM, Cohen C 2006 Crystal structure of apo-calmodulin bound to the first two IQ motifs of myosin V reveals essential recognition features. Proceedings of the National Academy of Sciences, USA 103, 19326–19331. 10.1073/pnas.0609436103 PMC1687203 17151196

[CIT0041] Huang Z, Van Houten J, Gonzalez G, Xiao H, van der Knaap E 2013 Genome-wide identification, phylogeny and expression analysis of *SUN*, *OFP* and *YABBY* gene family in tomato. Molecular Genetics and Genomics 288, 111–129. 23371549 10.1007/s00438-013-0733-0

[CIT0042] Hussey PJ, Ketelaar T, Deeks MJ 2006 Control of the actin cytoskeleton in plant cell growth. Annual Review of Plant Biology 57, 109–125. 10.1146/annurev.arplant.57.032905.105206 16669757

[CIT0043] Ivakov A, Persson S 2013 Plant cell shape: modulators and measurements. Frontiers in Plant Science 4, 439. 24312104 10.3389/fpls.2013.00439 PMC3832843

[CIT0044] Jacques E, Verbelen JP, Vissenberg K 2014 Review on shape formation in epidermal pavement cells of the Arabidopsis leaf. Functional Plant Biology 41, 914–921. 10.1071/FP13338 32481044

[CIT0045] Karimi M, Inzé D, Depicker A 2002 GATEWAY vectors for *Agrobacterium*-mediated plant transformation. Trends in Plant Science 7, 193–195. 11992820 10.1016/s1360-1385(02)02251-3

[CIT0046] Kelley LA, Mezulis S, Yates CM, Wass MN, Sternberg MJ 2015 The Phyre2 web portal for protein modeling, prediction and analysis. Nature Protocols 10, 845–858. 25950237 10.1038/nprot.2015.053 PMC5298202

[CIT0047] Kerstetter RA, Poethig RS 1998 The specification of leaf identity during shoot development. Annual Review of Cell and Developmental Biology 14, 373–398. 10.1146/annurev.cellbio.14.1.373 9891788

[CIT0048] Kudla J, Becker D, Grill E, Hedrich R, Hippler M, Kummer U, Parniske M, Romeis T, Schumacher K 2018 Advances and current challenges in calcium signaling. New Phytologist 218, 414–431. 29332310 10.1111/nph.14966

[CIT0049] Lee YK, Kim GT, Kim IJ, Park J, Kwak SS, Choi G, Chung WI 2006 *LONGIFOLIA1* and *LONGIFOLIA2*, two homologous genes, regulate longitudinal cell elongation in *Arabidopsis*. Development 133, 4305–4314. 17038516 10.1242/dev.02604

[CIT0050] Levy M, Wang Q, Kaspi R, Parrella MP, Abel S 2005 Arabidopsis IQD1, a novel calmodulin-binding nuclear protein, stimulates glucosinolate accumulation and plant defense. The Plant Journal 43, 79–96. 15960618 10.1111/j.1365-313X.2005.02435.x

[CIT0051] Liang H, Zhang Y, Martinez P, Rasmussen CG, Xu T, Yang Z 2018 The microtubule-associated protein IQ67 DOMAIN5 modulates microtubule dynamics and pavement cell shape. Plant Physiology 177, 1555–1568. 29976837 10.1104/pp.18.00558 PMC6084666

[CIT0052] Lin D, Cao L, Zhou Z, Zhu L, Ehrhardt D, Yang Z, Fu Y 2013 Rho GTPase signaling activates microtubule severing to promote microtubule ordering in Arabidopsis. Current Biology 23, 290–297. 23394835 10.1016/j.cub.2013.01.022

[CIT0053] Lincoln C, Britton JH, Estelle M 1990 Growth and development of the axr1 mutants of Arabidopsis. The Plant Cell 2, 1071–1080. 1983791 10.1105/tpc.2.11.1071 PMC159955

[CIT0054] Liu J, Chen J, Zheng X, et al 2017 GW5 acts in the brassinosteroid signalling pathway to regulate grain width and weight in rice. Nature Plants 3, 17043. 28394310 10.1038/nplants.2017.43

[CIT0055] Liu Z, Persson S, Zhang Y 2015 The connection of cytoskeletal network with plasma membrane and the cell wall. Journal of Integrative Plant Biology 57, 330–340. 25693826 10.1111/jipb.12342 PMC4405036

[CIT0056] Liu Z, Schneider R, Kesten C, Zhang Y, Somssich M, Zhang Y, Fernie AR, Persson S 2016 Cellulose–microtubule uncoupling proteins prevent lateral displacement of microtubules during cellulose synthesis in Arabidopsis. Developmental Cell 38, 305–315. 27477947 10.1016/j.devcel.2016.06.032

[CIT0057] Lloyd C, Hussey P 2001 Microtubule-associated proteins in plants—why we need a MAP. Nature Reviews. Molecular Cell Biology 2, 40–47. 11413464 10.1038/35048005

[CIT0058] Locascio A, Blázquez MA, Alabadí D 2013 Dynamic regulation of cortical microtubule organization through prefoldin–DELLA interaction. Current Biology 23, 804–809. 23583555 10.1016/j.cub.2013.03.053

[CIT0059] Lu Q, Li J, Ye F, Zhang M 2015 Structure of myosin-1c tail bound to calmodulin provides insights into calcium-mediated conformational coupling. Nature Structural & Molecular Biology 22, 81–88. 10.1038/nsmb.2923 25437912

[CIT0060] Majda M, Grones P, Sintorn IM, et al 2017 Mechanochemical polarization of contiguous cell walls shapes plant pavement cells. Developmental Cell 43, 290–304.e4. 29112850 10.1016/j.devcel.2017.10.017

[CIT0061] Marc J, Granger CL, Brincat J, Fisher DD, Kao Th, McCubbin AG, Cyr RJ 1998 A GFP–MAP4 reporter gene for visualizing cortical microtubule rearrangements in living epidermal cells. The Plant Cell 10, 1927–1940. 9811799 10.1105/tpc.10.11.1927 PMC143949

[CIT0062] Möller B, Glaß M, Misiak D, Posch S 2016 MiToBo—a toolbox for image processing and analysis. Journal of Open Research Software 4, e17.

[CIT0063] Möller B, Poeschl Y, Plötner R, Bürstenbinder K 2017 PaCeQuant: a tool for high-throughput quantification of pavement cell shape characteristics. Plant Physiology 175, 998–1017. 28931626 10.1104/pp.17.00961 PMC5664455

[CIT0064] Morejohn LC, Bureau TE, Molè-Bajer J, Bajer AS, Fosket DE 1987 Oryzalin, a dinitroaniline herbicide, binds to plant tubulin and inhibits microtubule polymerization in vitro. Planta 172, 252–264. 24225878 10.1007/BF00394595

[CIT0065] Mukhtar MS, Carvunis AR, Dreze M, et al 2011 Independently evolved virulence effectors converge onto hubs in a plant immune system network. Science 333, 596–601. 21798943 10.1126/science.1203659 PMC3170753

[CIT0066] Oda Y 2018 Emerging roles of cortical microtubule–membrane interactions. Journal of Plant Research 131, 5–14. 29170834 10.1007/s10265-017-0995-4

[CIT0067] Pan YP, Liang XJ, Gao ML, Liu HQ, Meng HW, Weng YQ, Cheng ZH 2018 Round fruit shape in WI7239 cucumber is controlled by two interacting quantitative trait loci with one putatively encoding a tomato SUN homolog. Theoretical and Applied Genetics 130, 573–586. 10.1007/s00122-016-2836-6 27915454

[CIT0068] Paredez AR, Somerville CR, Ehrhardt DW 2006 Visualization of cellulose synthase demonstrates functional association with microtubules. Science 312, 1491–1495. 16627697 10.1126/science.1126551

[CIT0069] Poethig RS 1997 Leaf morphogenesis in flowering plants. The Plant Cell 9, 1077–1087. 9254931 10.1105/tpc.9.7.1077 PMC156981

[CIT0070] Qian P, Hou S, Guo G 2009 Molecular mechanisms controlling pavement cell shape in Arabidopsis leaves. Plant Cell Reports 28, 1147–1157. 19529941 10.1007/s00299-009-0729-8

[CIT0071] Sampathkumar A, Krupinski P, Wightman R, Milani P, Berquand A, Boudaoud A, Hamant O, Jönsson H, Meyerowitz EM 2014 Subcellular and supracellular mechanical stress prescribes cytoskeleton behavior in Arabidopsis cotyledon pavement cells. eLife 3, e01967. 24740969 10.7554/eLife.01967 PMC3985187

[CIT0072] Saunders MJ, Hepler PK 1981 Localization of membrane-associated calcium following cytokinin treatment in Funaria using chlorotetracycline. Planta 152, 272–281. 24302427 10.1007/BF00385156

[CIT0073] Schindelin J, Arganda-Carreras I, Frise E, et al 2012 Fiji: an open-source platform for biological-image analysis. Nature Methods 9, 676–682. 22743772 10.1038/nmeth.2019 PMC3855844

[CIT0074] Schneider R, Persson S 2015 Connecting two arrays: the emerging role of actin–microtubule cross-linking motor proteins. Frontiers in Plant Science 6, 415. 26082793 10.3389/fpls.2015.00415 PMC4451249

[CIT0075] Seagull RW 1986 Changes in microtubule organization and wall microfibril orientation during in vitro cotton fiber development—an immunofluorescent study. Canadian Journal of Botany 64, 1373–1381.

[CIT0076] Sedbrook JC 2004 MAPs in plant cells: delineating microtubule growth dynamics and organization. Current Opinion in Plant Biology 7, 632–640. 15491911 10.1016/j.pbi.2004.09.017

[CIT0077] Sheahan MB, Staiger CJ, Rose RJ, McCurdy DW 2004 A green fluorescent protein fusion to actin-binding domain 2 of Arabidopsis fimbrin highlights new features of a dynamic actin cytoskeleton in live plant cells. Plant Physiology 136, 3968–3978. 15557099 10.1104/pp.104.049411 PMC535829

[CIT0078] Shibaoka H 1994 Plant hormone-induced changes in the orientation of cortical microtubules—alterations in the cross-linking between microtubules and the plasma-membrane. Annual Review of Plant Physiology and Plant Molecular Biology 45, 527–544.

[CIT0079] Sugiyama Y, Wakazaki M, Toyooka K, Fukuda H, Oda Y 2017 A novel plasma membrane-anchored protein regulates xylem cell-wall deposition through microtubule-dependent lateral inhibition of Rho GTPase domains. Current Biology 27, 2522–2528. 28803875 10.1016/j.cub.2017.06.059

[CIT0080] Takatani S, Hirayama T, Hashimoto T, Takahashi T, Motose H 2015 Abscisic acid induces ectopic outgrowth in epidermal cells through cortical microtubule reorganization in *Arabidopsis thaliana*. Scientific Reports 5, 11364. 26068445 10.1038/srep11364 PMC4464343

[CIT0081] Tsukaya H 1995 Developmental genetics of leaf morphogenesis in dicotyledonous plants. Journal of Plant Research 108, 407–416.

[CIT0082] Tsukaya H 2002 Leaf development. The Arabidopsis Book 1, e0072. 22303217 10.1199/tab.0072 PMC3243299

[CIT0083] Tsukaya H, Tsuge T, Uchimiya H 1994 The cotyledon—a superior system for studies of leaf development. Planta 195, 309–312.

[CIT0084] Vanneste S, Friml J 2013 Calcium: the missing link in auxin action. Plants 2, 650–675. 27137397 10.3390/plants2040650 PMC4844386

[CIT0085] Wang YS, Motes CM, Mohamalawari DR, Blancaflor EB 2004 Green fluorescent protein fusions to Arabidopsis fimbrin 1 for spatio-temporal imaging of F-actin dynamics in roots. Cell Motility and the Cytoskeleton 59, 79–93. 15362112 10.1002/cm.20024

[CIT0086] Wasteneys GO 2002 Microtubule organization in the green kingdom: chaos or self-order?Journal of Cell Science 115, 1345–1354. 11896182 10.1242/jcs.115.7.1345

[CIT0087] Wasteneys GO, Yang Z 2004 *a* The cytoskeleton becomes multidisciplinary. Plant Physiology 136, 3853–3854. 15591442 10.1104/pp.104.900130 PMC535818

[CIT0088] Wasteneys GO, Yang Z 2004 *b* New views on the plant cytoskeleton. Plant Physiology 136, 3884–3891. 15591446 10.1104/pp.104.900133 PMC535822

[CIT0089] Winter D, Vinegar B, Nahal H, Ammar R, Wilson GV, Provart NJ 2007 An ‘Electronic Fluorescent Pictograph’ browser for exploring and analyzing large-scale biological data sets. PLoS One 2, e718. 17684564 10.1371/journal.pone.0000718 PMC1934936

[CIT0090] Wood P 1984 Specific interaction of aniline blue with (1,3)-beta-d-glucan. Carbohydrate Polymers 4, 49–72.

[CIT0091] Xiao H, Jiang N, Schaffner E, Stockinger EJ, van der Knaap E 2008 A retrotransposon-mediated gene duplication underlies morphological variation of tomato fruit. Science 319, 1527–1530. 18339939 10.1126/science.1153040

[CIT0092] Yang BJ, Wendrich JR, De B, Weijers D, Xue HW 2018 OsIQD14 regulates grain shape through modulating the microtubule cytoskeleton. bioRxiv doi: 10.1101/275552. [Preprint.]

[CIT0093] Zhang C, Halsey LE, Szymanski DB 2011 The development and geometry of shape change in *Arabidopsis thaliana* cotyledon pavement cells. BMC Plant Biology 11, 27. 21284861 10.1186/1471-2229-11-27 PMC3042916

