# Supplementary material for: Microtubule-associated protein IQ67 DOMAIN5 regulates morphogenesis of leaf pavement cells in Arabidopsis thaliana
Source: J Exp Bot. 2018 Nov 8;70(2):529–43. doi: 10.1093/jxb/ery395 (PMC6322583; doi:10.1093/jxb/ery395)
Supplement: Supplementary Material [file ery395_suppl_supplementary-figures-s1-s11.pdf]

Supplementary Fig. S1.

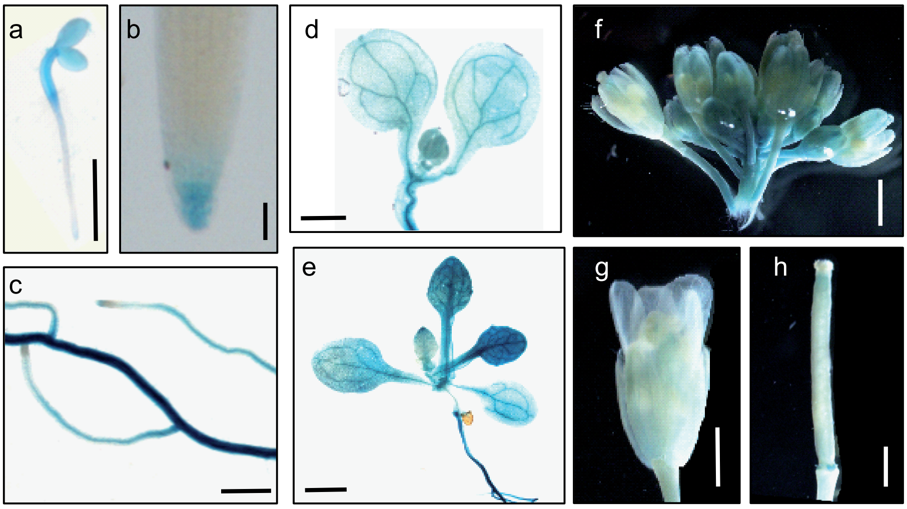

Supplementary Fig. S1.
IQD5 expression analysis in *pIQD5$_{long}$::GFP-GUS* reporter lines. Whole mount histochemical GUS staining of 2-day-old seedlings (a), in the primary root meristem (b), lateral roots (c) and cotyledons (d) of 5-day-old seedlings, in the shoot of 10-day-old seedlings (e), and in flower buds f), flowers (g) and siliques (h) of 5-week-old plants. Scale bars represent 1 mm (a, c-h) and 10 µm (b).

Supplementary Fig. S2.

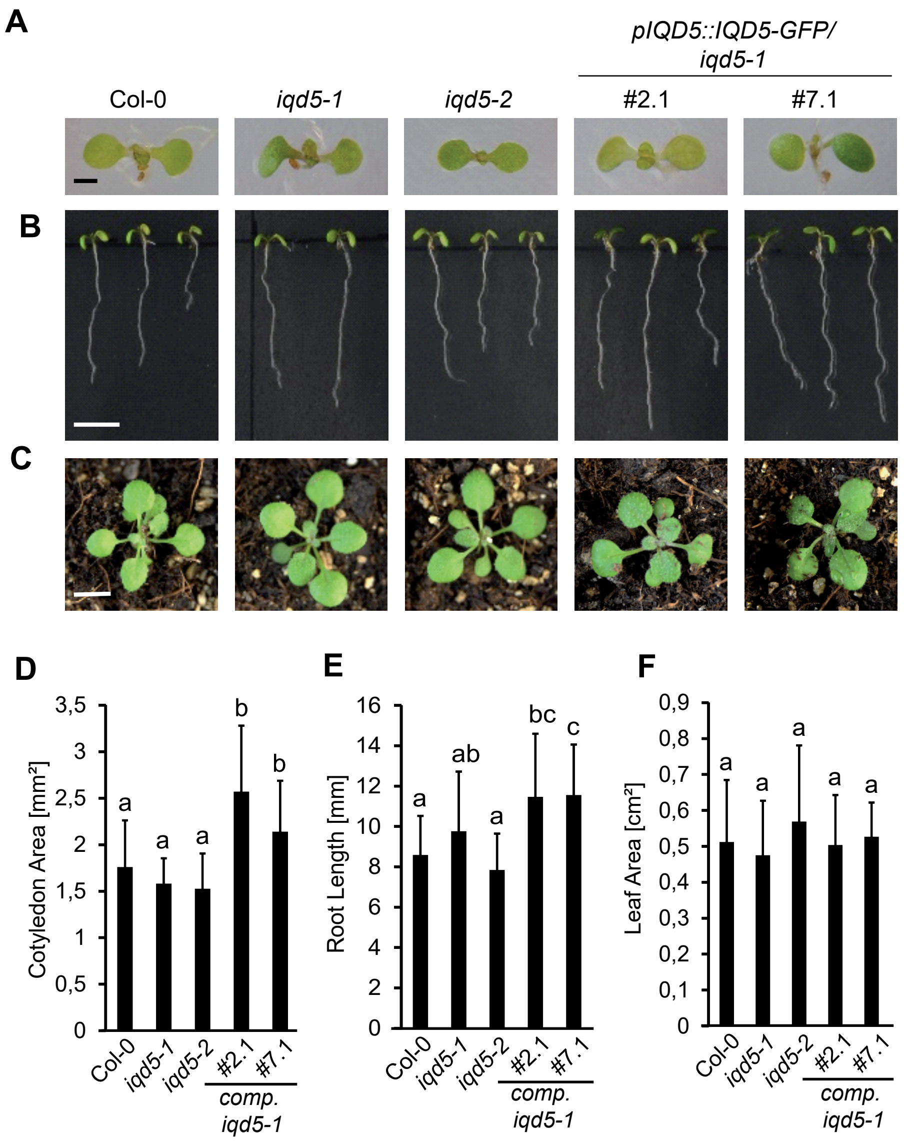

Supplementary Fig. S2.
Macroscopic analysis of growth parameters in wild type and *iqd5* mutants. Seedlings of wild type, the two *iqd5* mutant alleles *iqd5-1* and *iqd5-2*, and two independent *pIQD5::IQD5-GFP/iqd5-1* complementation lines grown under long-day conditions at 5 days after germination (A, B) and at 3 weeks after germination (C). Surface view of cotyledons (A) and side view of complete seedlings (B). Surface view of rosettes (C). Bars, 1 mm (A); 1 cm (B, C). Quantification of coteldon area (D) and root length (E) in seedlings shown in A and B. Quantification of leaf area (F) in seedlings shown in C. Data represent mean values ± standard deviation from n = 25-56 (D), n = 53-72 (E) and n = 30 (F) seedlings or plants. Different letters indicate statistically significant differences by one-way ANOVA; $p < 0.01$.

Fig. S3.

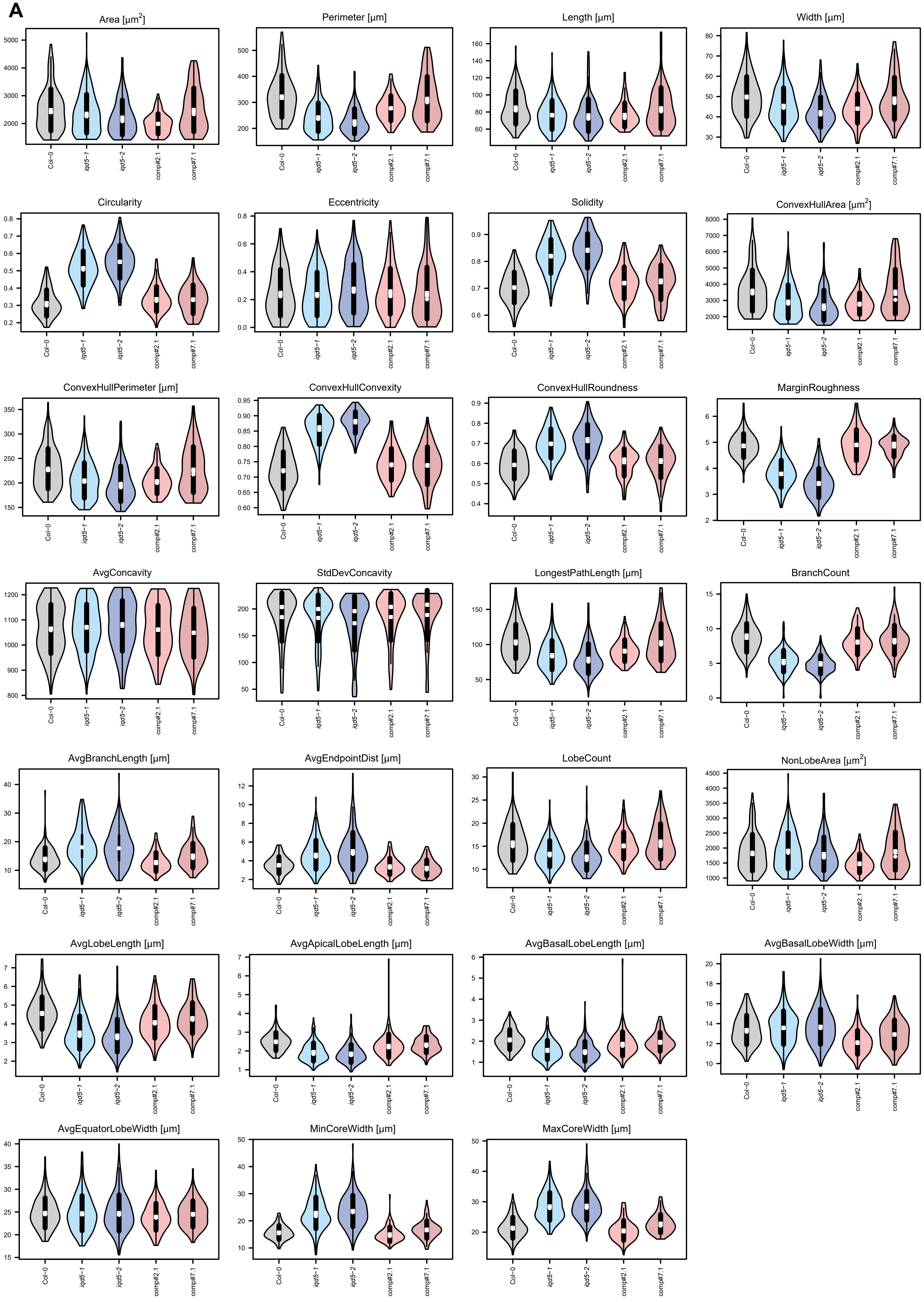
A
Area [μm2]
Perimeter [μm]
Length [μm]
Width [μm]
Circularity
Eccentricity
Solidity
ConvexHullArea [μm2]
ConvexHullPerimeter [μm]
ConvexHullConvexity
ConvexHullRoundness
MarginRoughness
AvgConcavity
StdDevConcavity
LongestPathLength [μm]
BranchCount
AvgBranchLength [μm]
AvgEndpointDist [μm]
LobeCount
NonLobeArea [μm2]
AvgLobeLength [μm]
AvgApicalLobeLength [μm]
AvgBasalLobeLength [μm]
AvgBasalLobeWidth [μm]
AvgEquatorLobeWidth [μm]
MinCoreWidth [μm]
MaxCoreWidth [μm]
Col−0
iqd5−1
iqd5−2
comp#2.1
comp#7.1

B

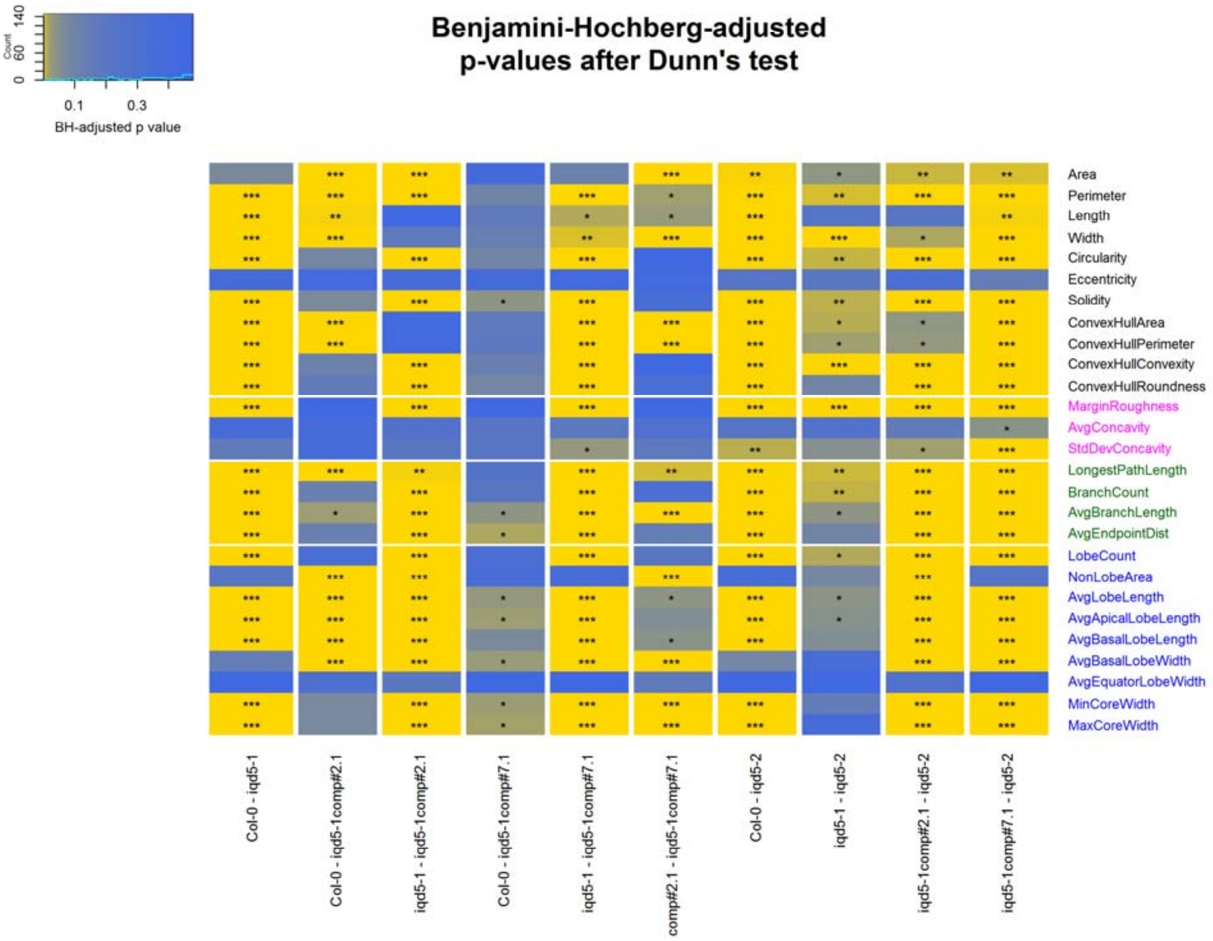

Supplementary Fig. S3.
Quantification and statistical analysis of PC shape features in 5-day-old seedlings of the wild type and *iqd5* mutants. Quantitative analysis of pavement cell shape features in 5-day-old seedlings of the wild type, *iqd5-1*, *iqd5-2* and two independent *pIQD5::IQD5-GFP/iqd5-1* complementation lines. Violin plots of all 27 features quantified with PaCeQuant (A). Statistical analysis from pairwise comparison between the analyzed genotypes (B). Shown are Benjamini-Hochberg-adjusted p-values after Dunn‘s pairwise test. Blue colors represent p-values close to 1, yellow colors represent values close to 0. Stars indicate statistically significant differences (* padj < 0.05, ** padj < 0.01, *** padj < 0.005).

Supplementary Fig. S4

A

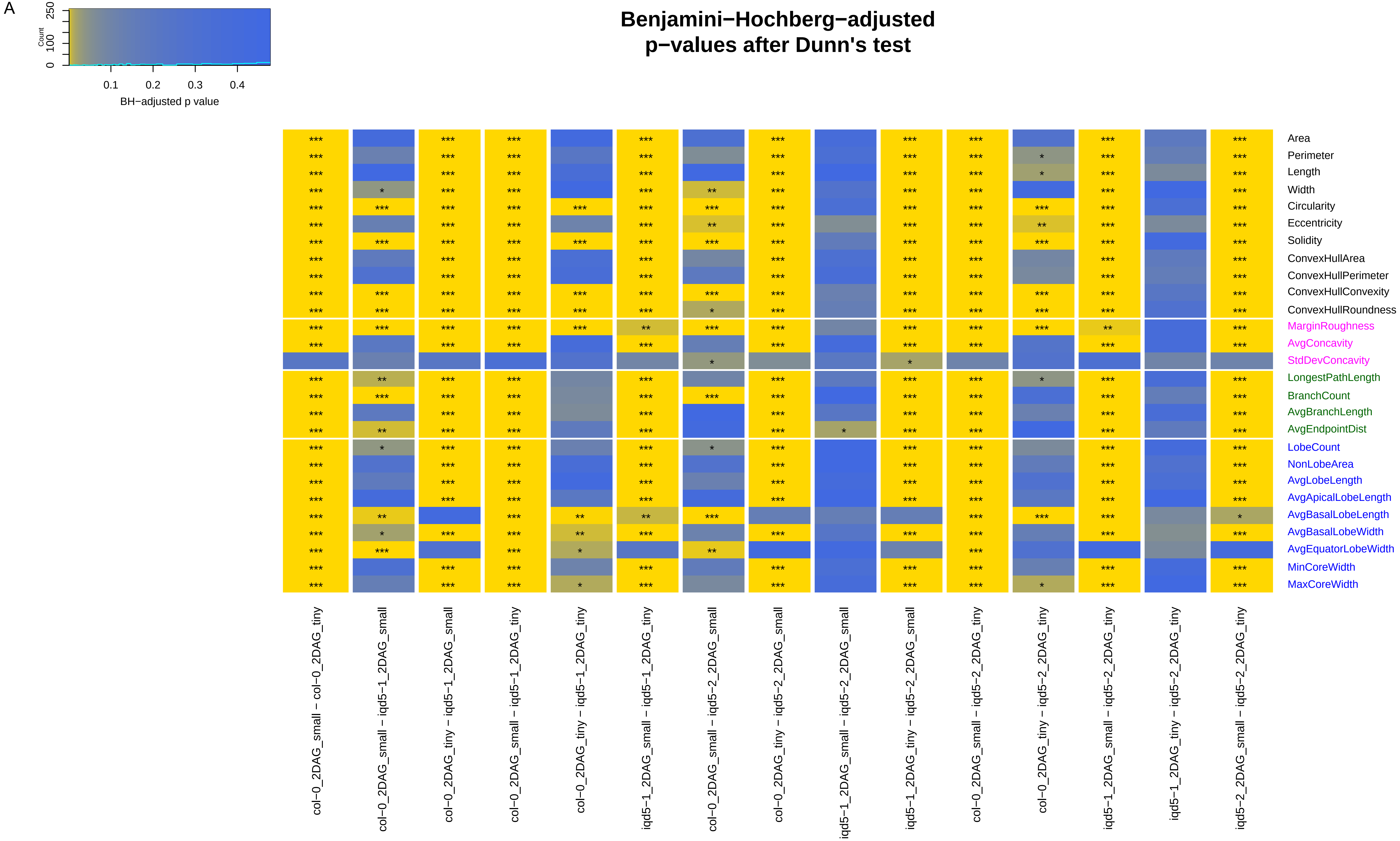

B

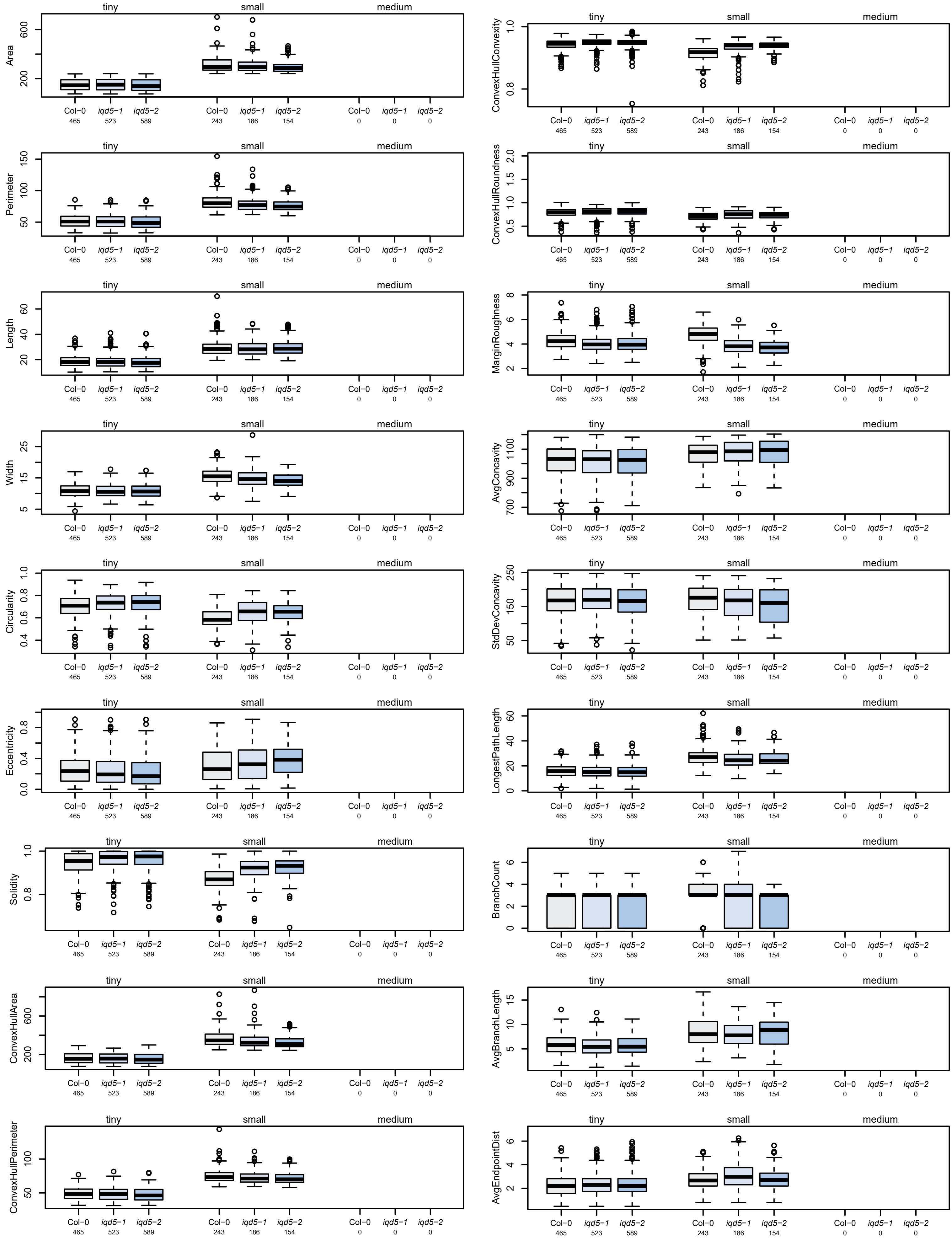

tiny
small
medium
Col−0
iqd5−1
iqd5−2
465
523
589
243
186
154
0
Area
Perimeter
Length
Width
Circularity
Eccentricity
Solidity
ConvexHullArea
ConvexHullPerimeter
ConvexHullConvexity
ConvexHullRoundness
MarginRoughness
AvgConcavity
StdDevConcavity
LongestPathLength
BranchCount
AvgBranchLength
AvgEndpointDist

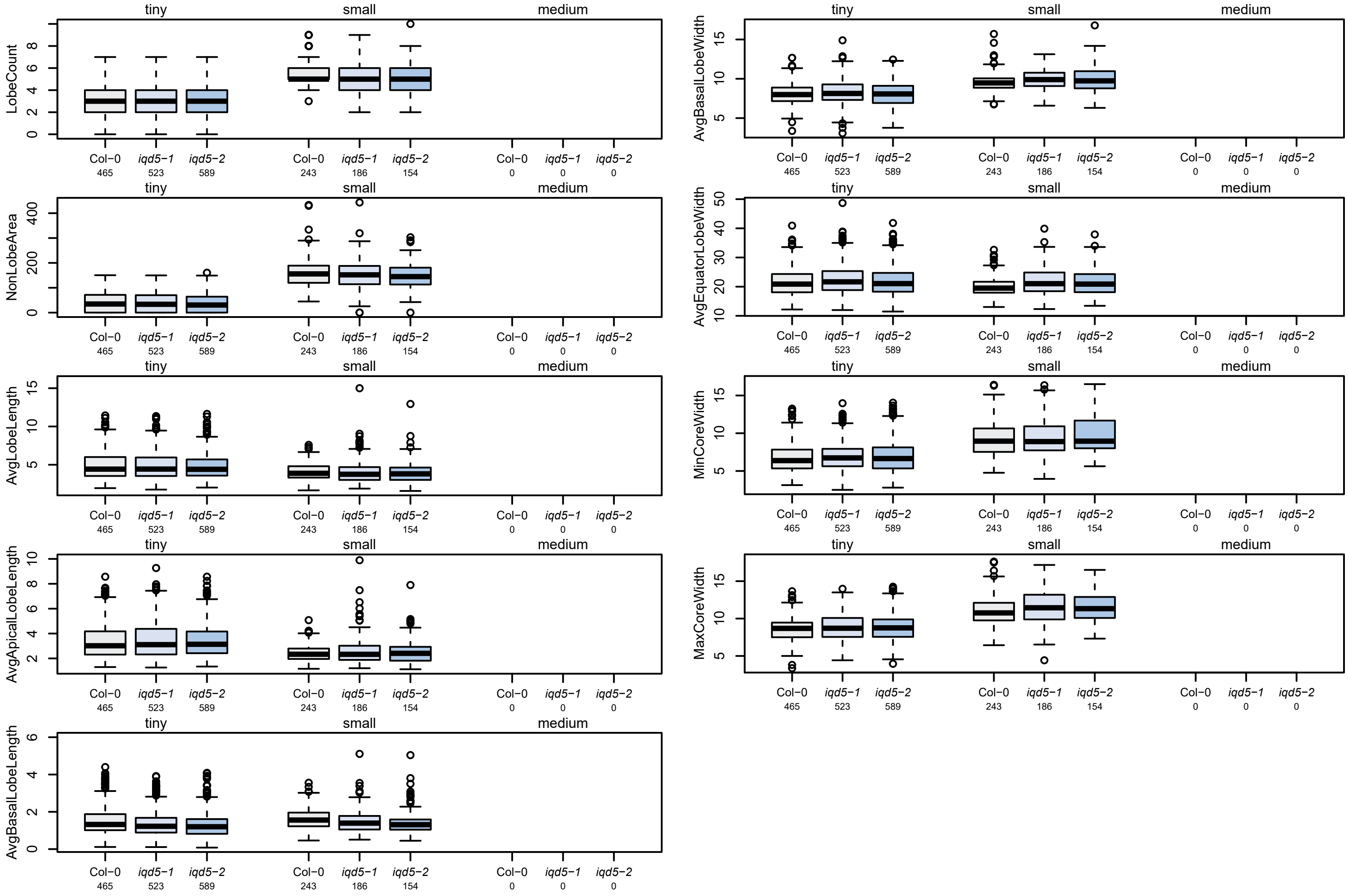

Supplementary Fig. S4.
Quantification and statistical analysis of PC shape in cotyledons at 2 DAG. Cells were grouped into tiny ($t_{tiny} \leq 240\ \mu m^2$) and small ($t_s \leq 1{,}400\ \mu m^2$) sized populations. Statistical analysis from pairwise comparisons within the three genotypes (Col-0, *iqd5-1* and *iqd5-2*) and the two size categories (tiny, small) (A). Quantification of shape features (B). Results are medians, boxes range from first to third quartile.

Supplementary Fig. S5

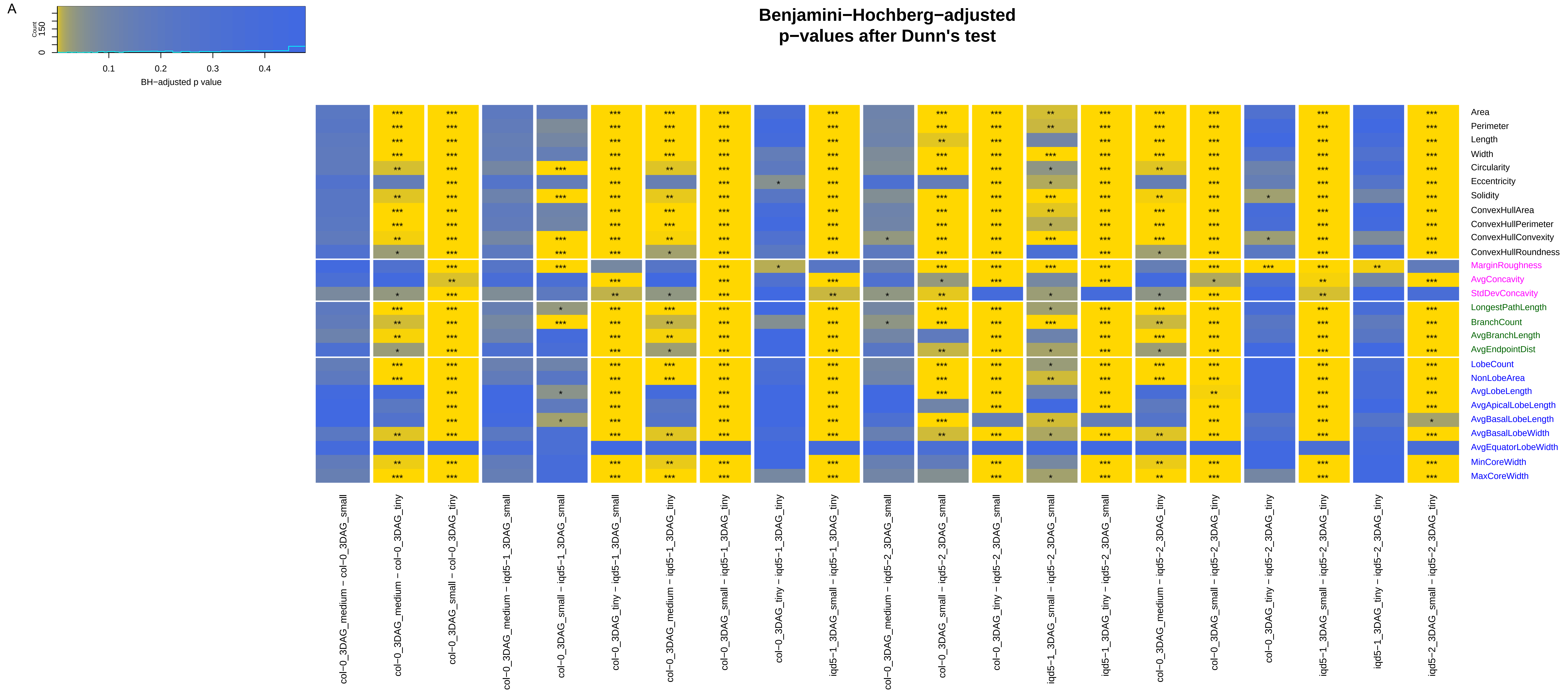

B

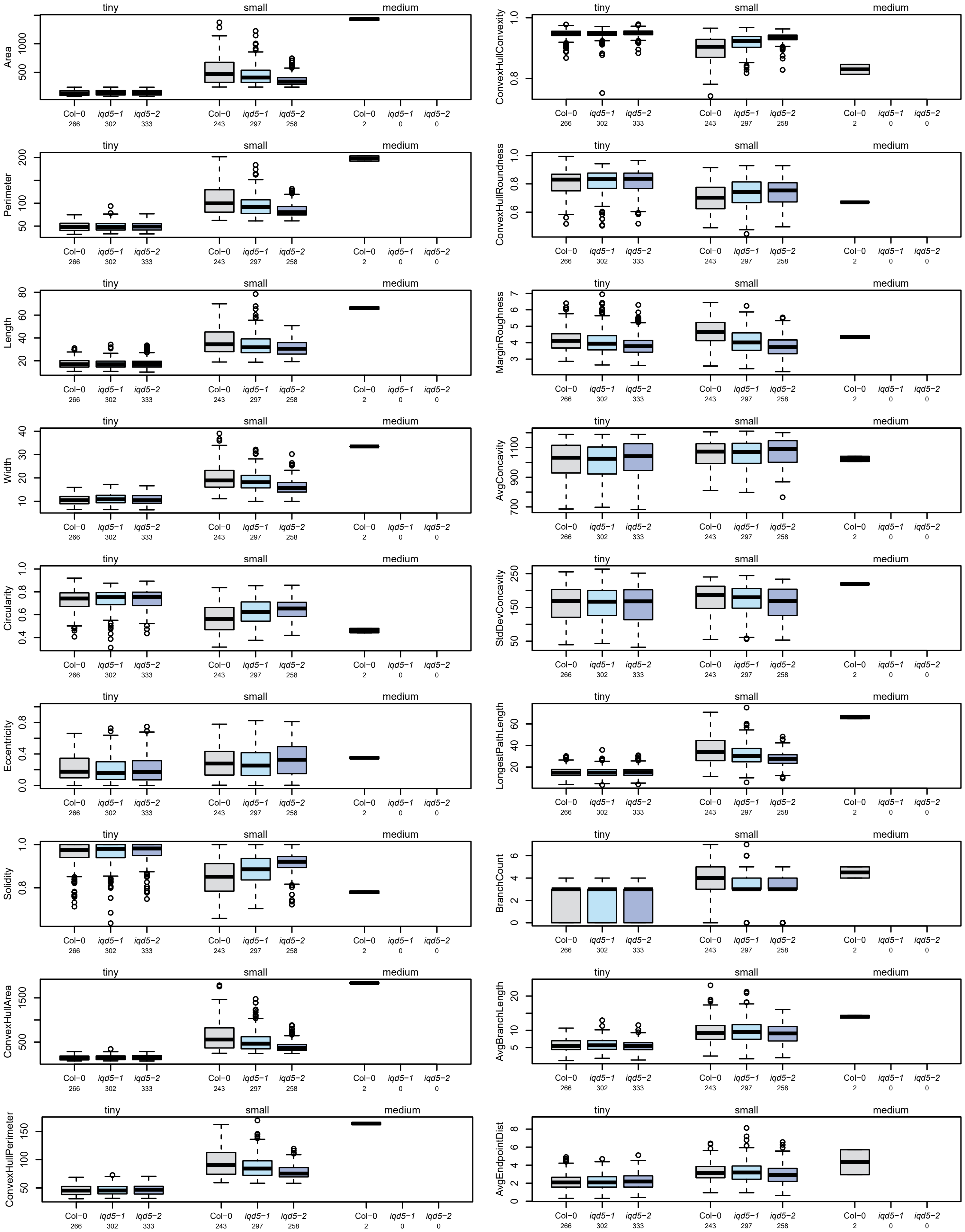

tiny
small
medium
Area
ConvexHullConvexity
Perimeter
ConvexHullRoundness
Length
MarginRoughness
Width
AvgConcavity
Circularity
StdDevConcavity
Eccentricity
LongestPathLength
Solidity
BranchCount
ConvexHullArea
AvgBranchLength
ConvexHullPerimeter
AvgEndpointDist
Col−0
iqd5−1
iqd5−2
266
302
333
243
297
258
2
0
0

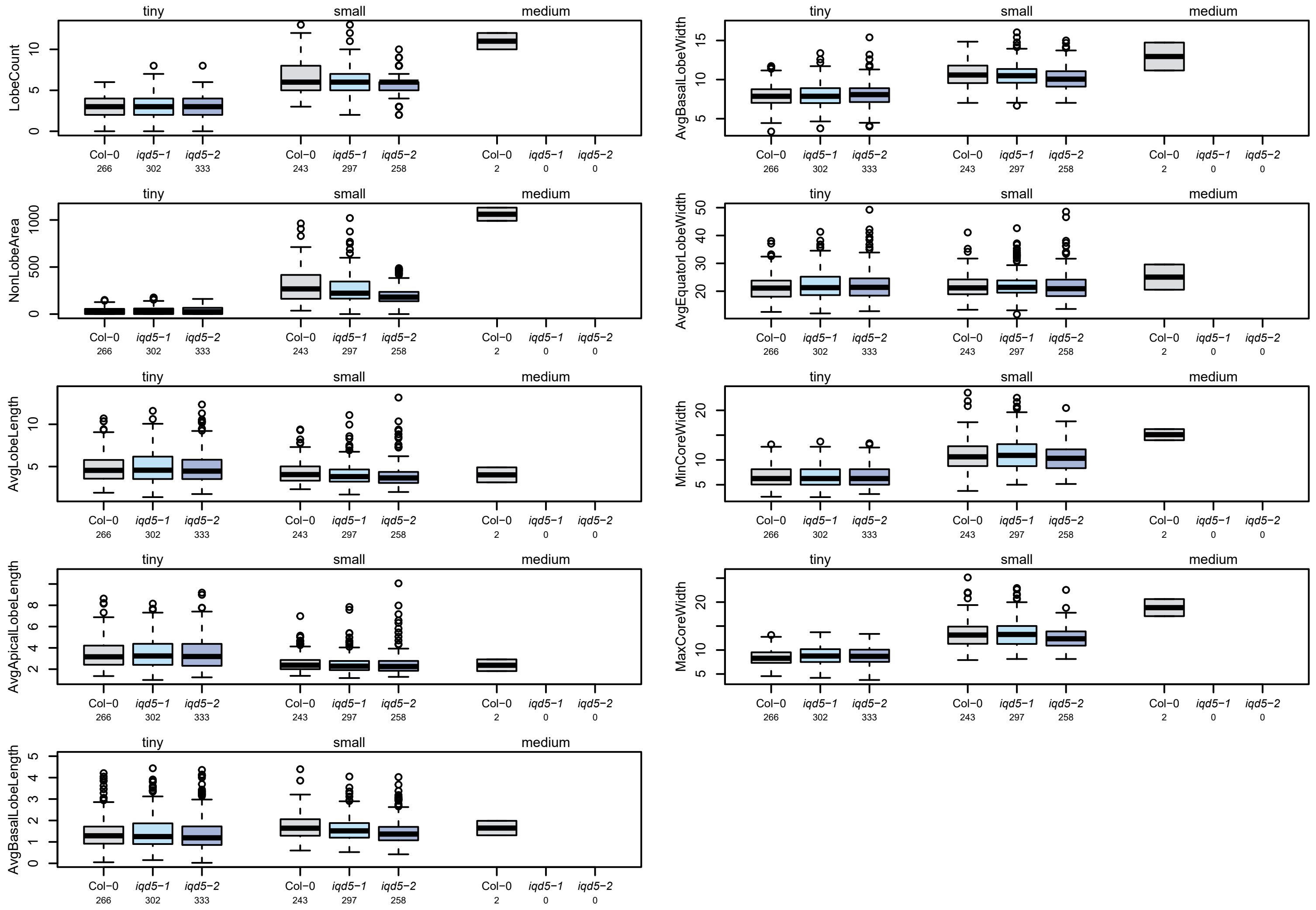

Supplementary Fig. S5.

Quantification and statistical analysis of PC shape in cotyledons at 3 DAG. Cells were grouped into tiny (t_{tiny} ≤ 240 μm^2), small (t_s ≤ 1,400 μm^2) and medium (t_m ≤ 4,042 μm^2) sized populations. Statistical analysis from pairwise comparisons within the three genotypes (Col-0, *iqd5-1* and *iqd5-2*) and the three size categories (tiny, small, medium) (A). Note that only two medium-sized cells were detected in Col-0, and no medium-sized cells were present in the two *iqd5* mutants.Quantification of shape features (B). Results are medians, boxes range from first to third quartile.

Supplementary Fig. S6

A

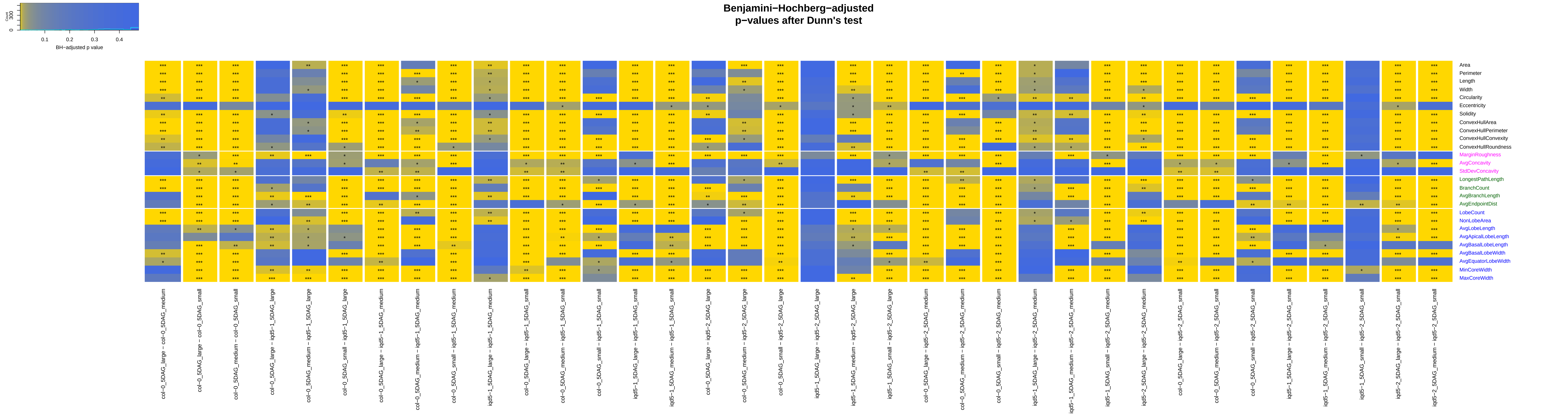

B

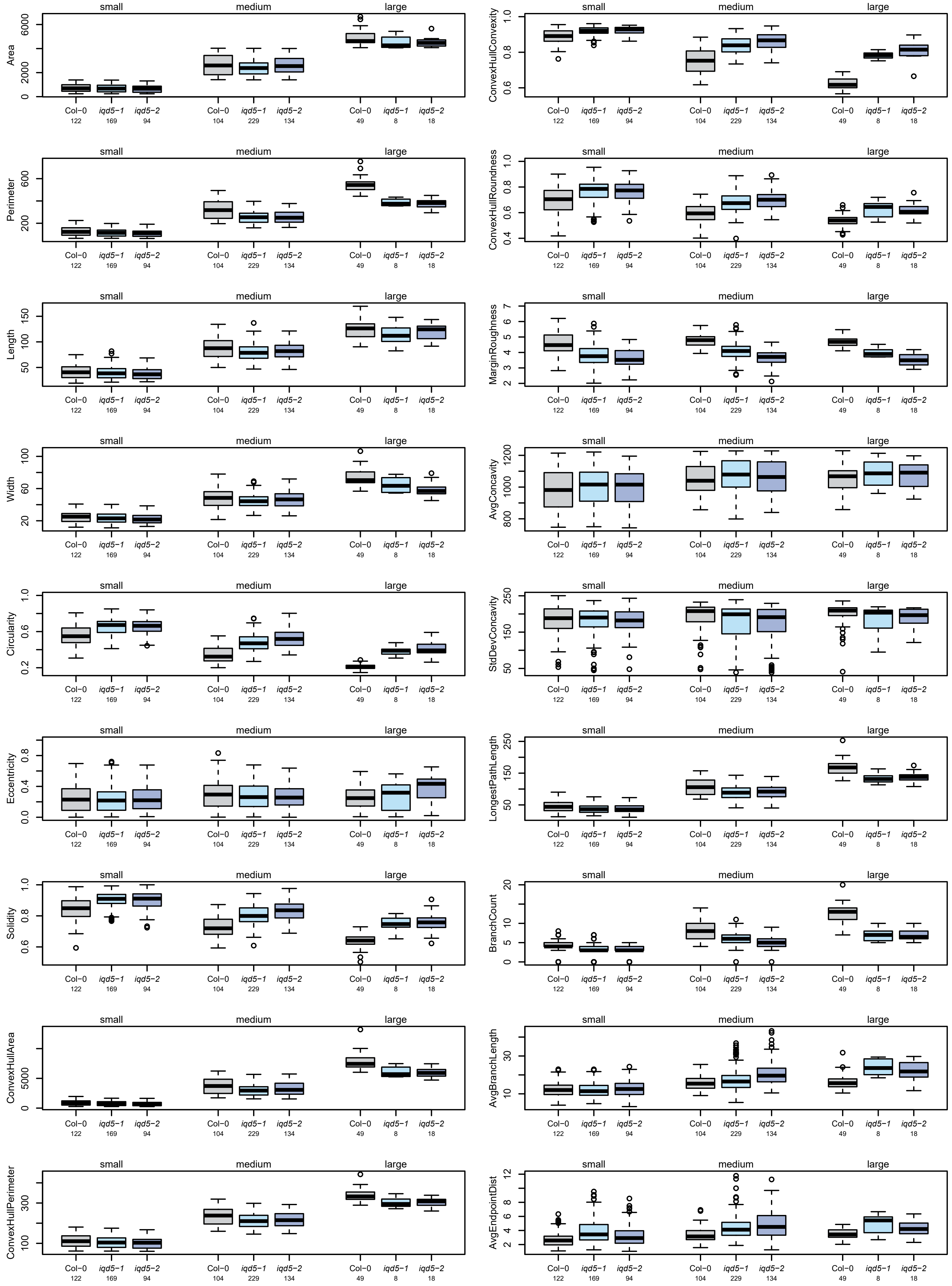
small
medium
large
Area
ConvexHullConvexity
Perimeter
ConvexHullRoundness
Length
MarginRoughness
Width
AvgConcavity
Circularity
StdDevConcavity
Eccentricity
LongestPathLength
Solidity
BranchCount
ConvexHullArea
AvgBranchLength
ConvexHullPerimeter
AvgEndpointDist
Col-0
iqd5-1
iqd5-2
122
169
94
104
229
134
49
8
18

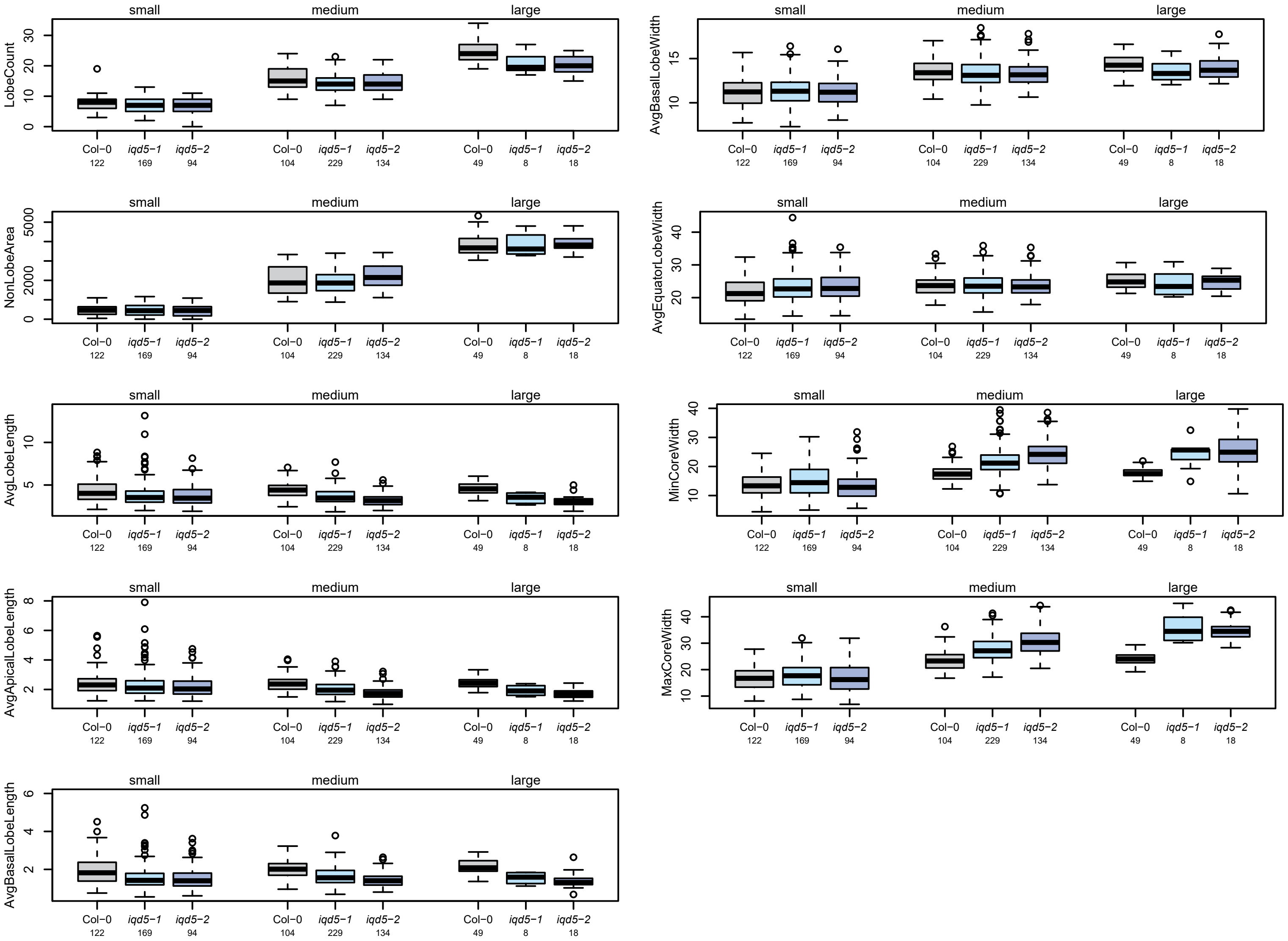

Supplementary Fig. S6.

Quantification and statistical analysis of PC shape in cotyledons at 5 DAG. Cells were grouped into small ($t_s \leq 1{,}400\ \mu m^2$), medium ($t_m \leq 4{,}042\ \mu m^2$) and large ($t_l > 4{,}042\ \mu m^2$) sized populations. Statistical analysis from pairwise comparisons within the three genotypes (Col-0, *iqd5-1* and *iqd5-2*) and the three size categories (small, medium, large) (A). Quantification of shape features (B). Results are medians, boxes range from first to third quartile.

Supplementary Fig. S7

A

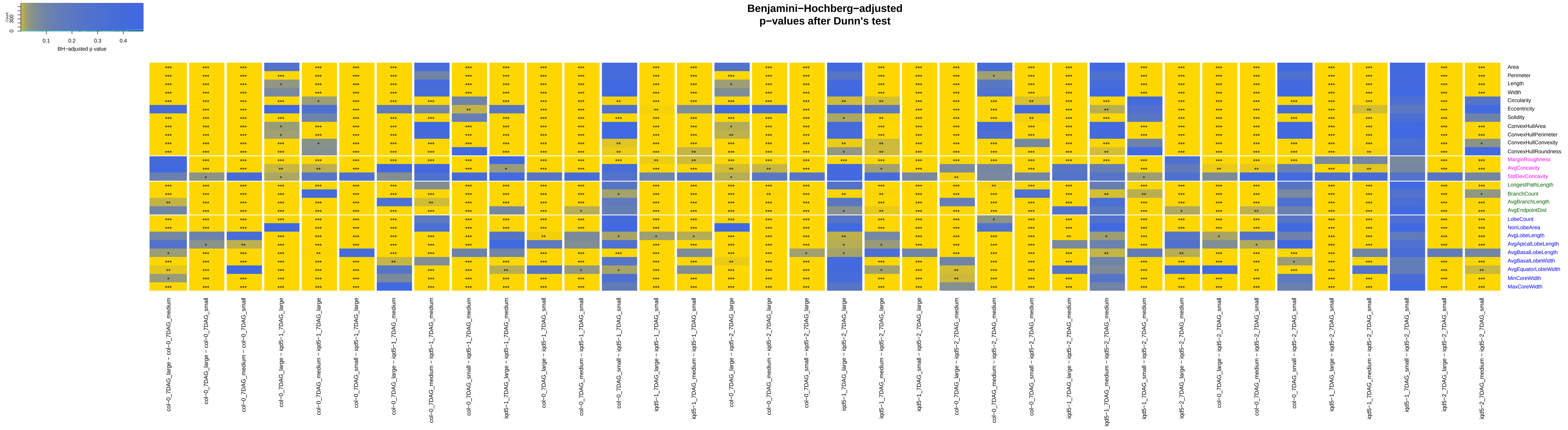

B

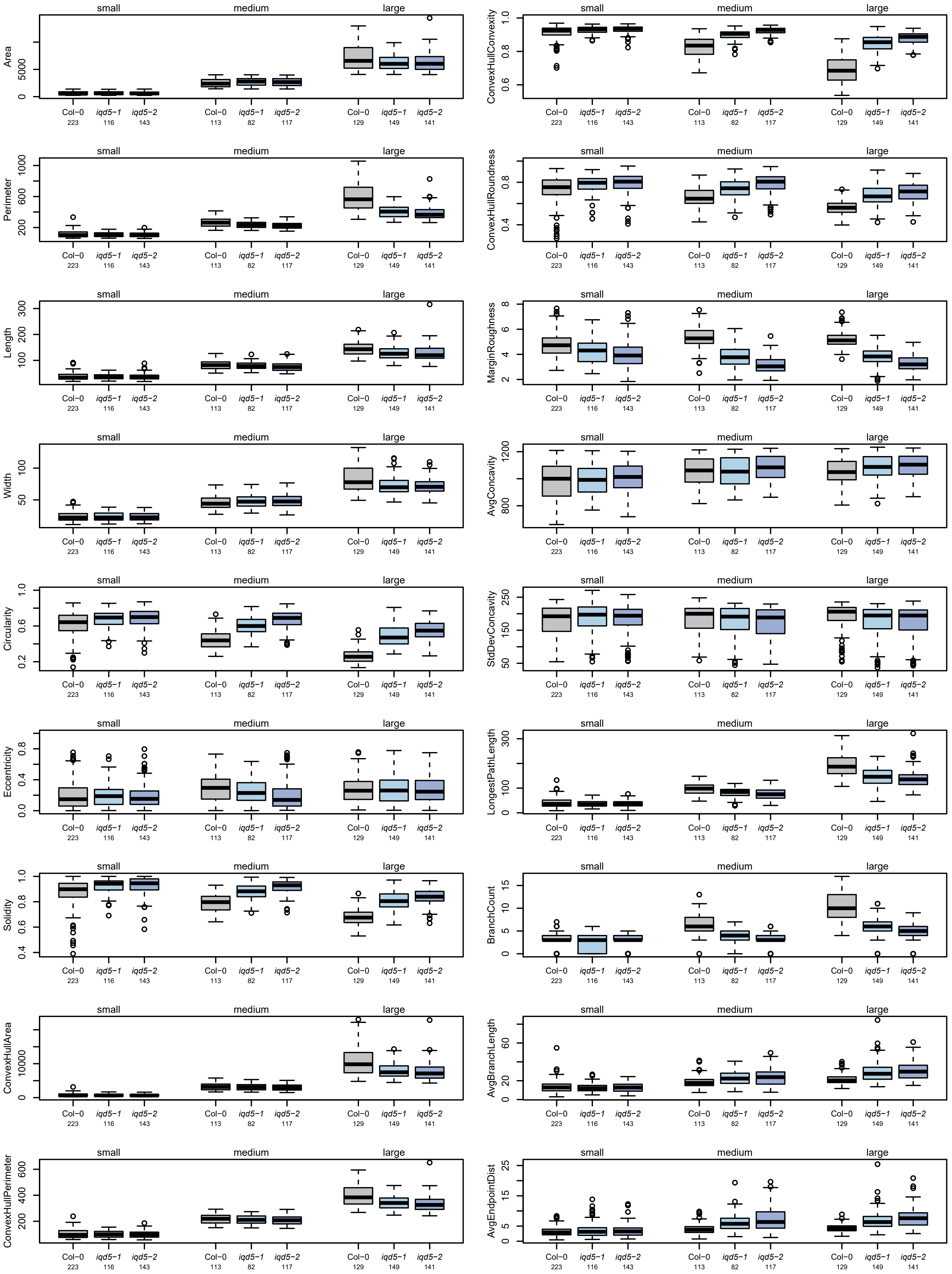
small
medium
large
Col-0
iqd5-1
iqd5-2
223
116
143
113
82
117
129
149
141
Area
ConvexHullConvexity
Perimeter
ConvexHullRoundness
Length
MarginRoughness
Width
AvgConcavity
Circularity
StdDevConcavity
Eccentricity
LongestPathLength
Solidity
BranchCount
ConvexHullArea
AvgBranchLength
ConvexHullPerimeter
AvgEndpointDist

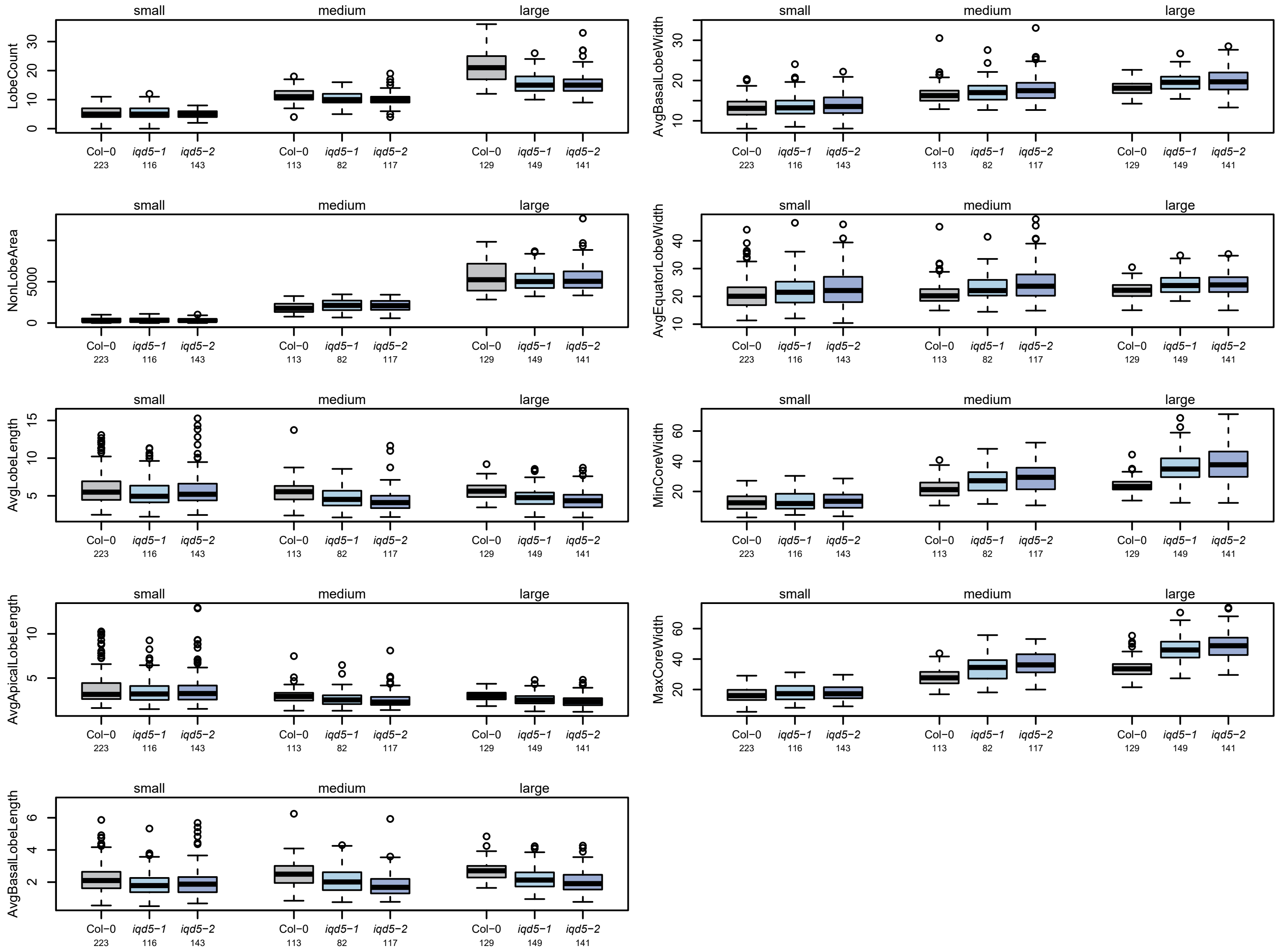

Supplementary Fig. S7.

Quantification and statistical analysis of PC shape in cotyledons at 7 DAG. Cells were grouped into small ($t_s \leq 1{,}400\ \mu m^2$), medium ($t_m \leq 4{,}042\ \mu m^2$) and large ($t_l > 4{,}042\ \mu m^2$) sized populations. Statistical analysis from pairwise comparisons within the three genotypes (Col-0, *iqd5-1* and *iqd5-2*) and the three size categories (small, medium, large) (A). Quantification of shape features (B). Results are medians, boxes range from first to third quartile.

Supplementary Fig. S8

A

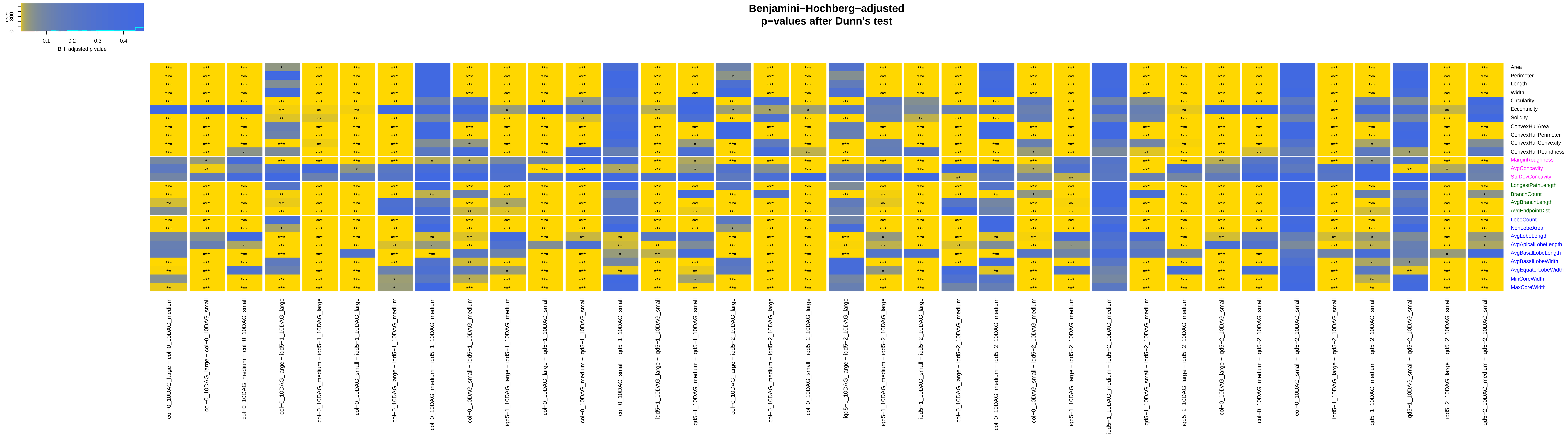

B

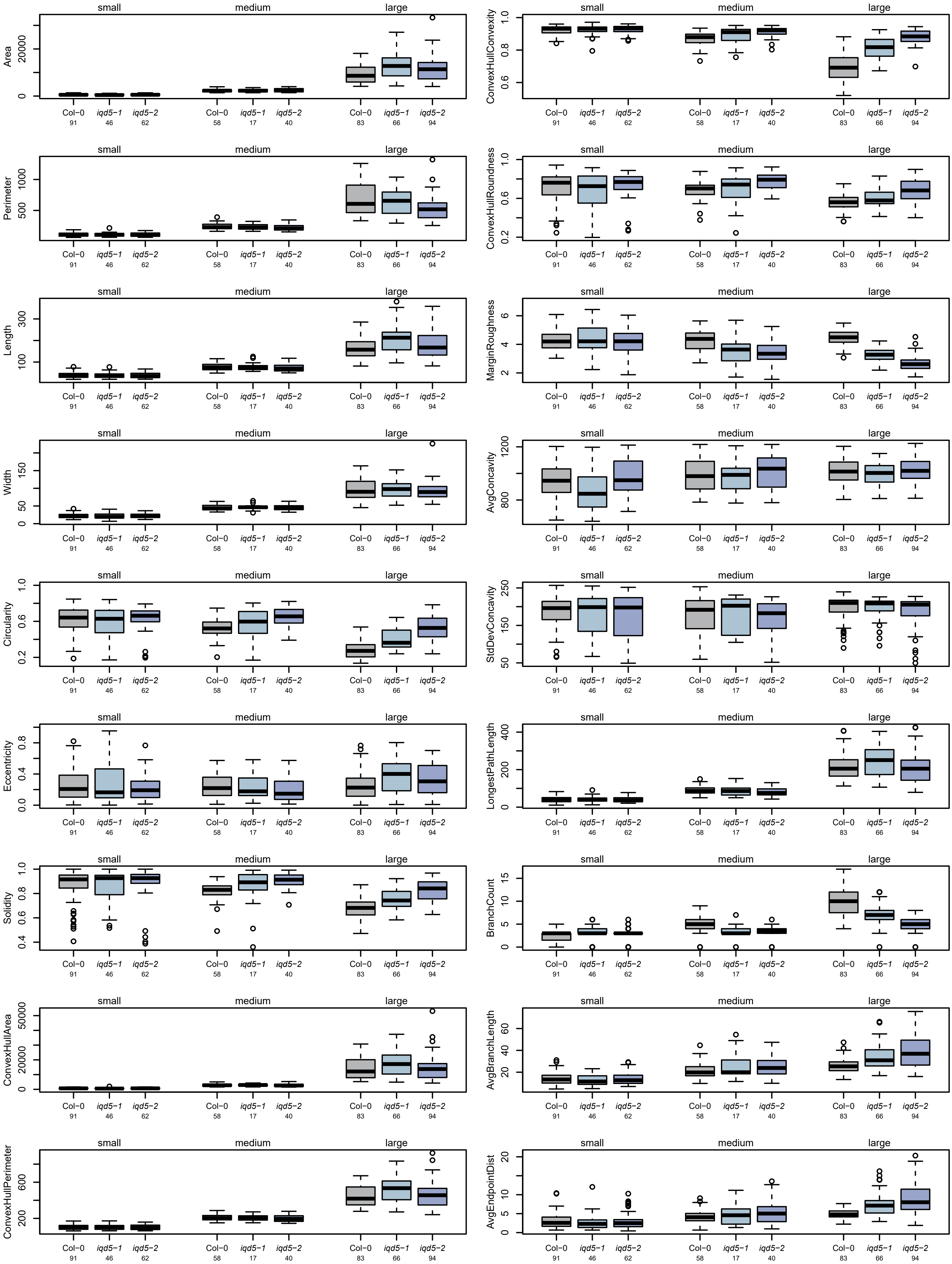

small
medium
large
Col-0
iqd5-1
iqd5-2
91
46
62
58
17
40
83
66
94
Area
ConvexHullConvexity
Perimeter
ConvexHullRoundness
Length
MarginRoughness
Width
AvgConcavity
Circularity
StdDevConcavity
Eccentricity
LongestPathLength
Solidity
BranchCount
ConvexHullArea
AvgBranchLength
ConvexHullPerimeter
AvgEndpointDist

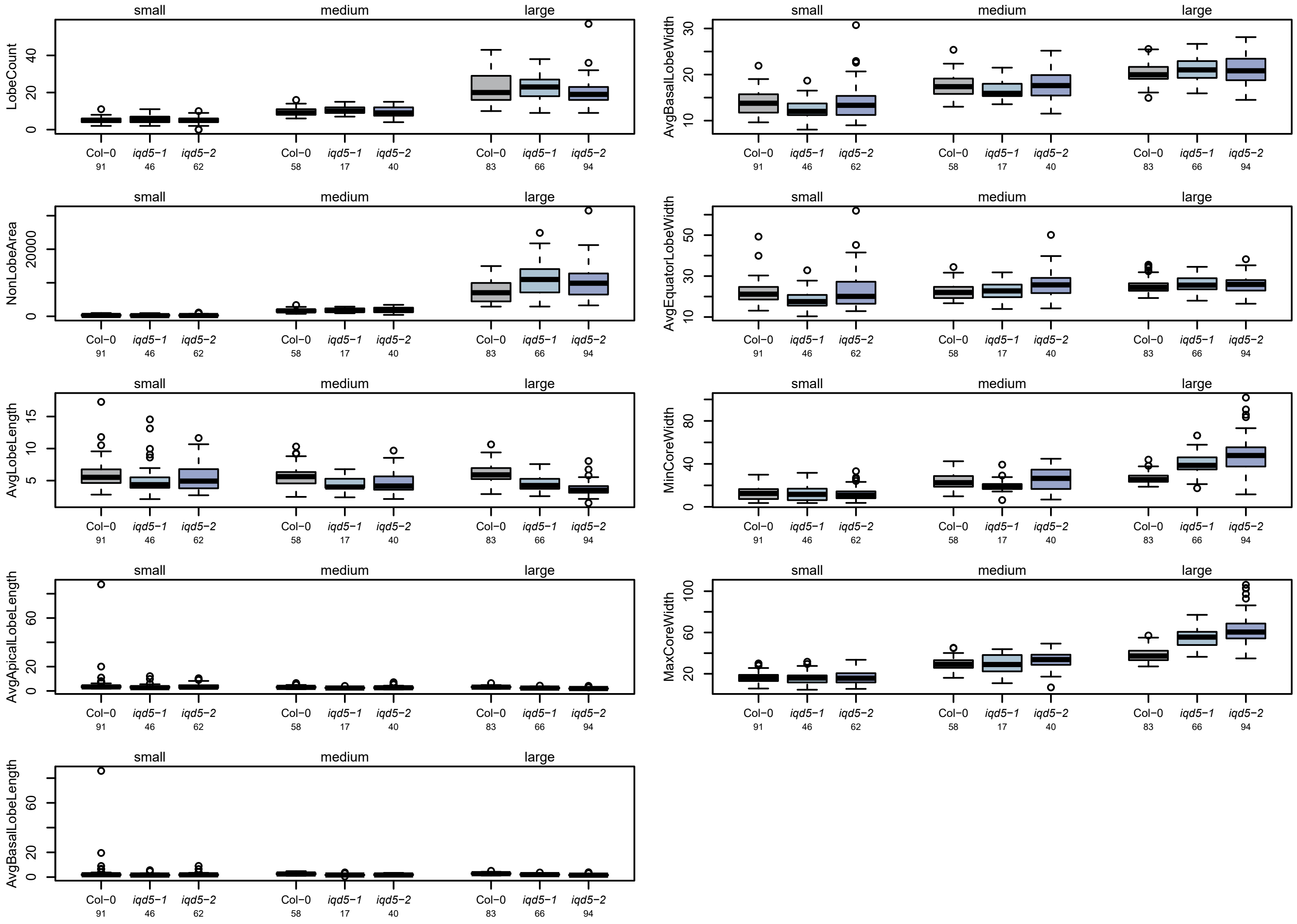

Supplementary Fig. S8.

Quantification and statistical analysis of PC shape in cotyledons at 10 DAG. Cells were grouped into small ($t_s \leq 1{,}400\ \mu m^2$), medium ($t_m \leq 4{,}042\ \mu m^2$) and large ($t_l > 4{,}042\ \mu m^2$) sized populations. Statistical analysis from pairwise comparisons within the three genotypes (Col-0, *iqd5-1* and *iqd5-2*) and the three size categories (small, medium, large) (A). Quantification of shape features (B). Results are medians, boxes range from first to third quartile.

Fig. S9.

A

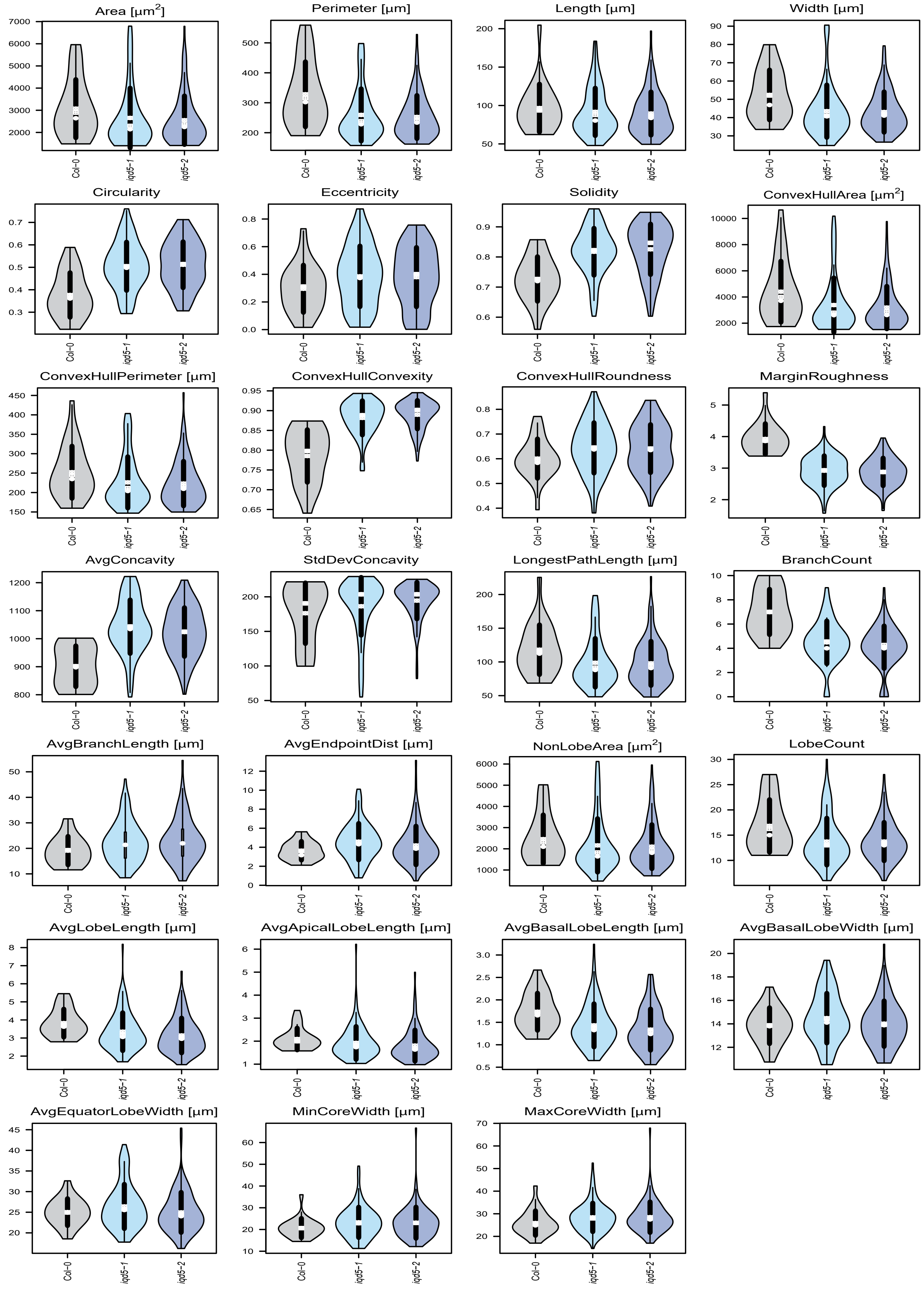

Area [μm²]
Perimeter [μm]
Length [μm]
Width [μm]
Circularity
Eccentricity
Solidity
ConvexHullArea [μm²]
ConvexHullPerimeter [μm]
ConvexHullConvexity
ConvexHullRoundness
MarginRoughness
AvgConcavity
StdDevConcavity
LongestPathLength [μm]
BranchCount
AvgBranchLength [μm]
AvgEndpointDist [μm]
NonLobeArea [μm²]
LobeCount
AvgLobeLength [μm]
AvgApicalLobeLength [μm]
AvgBasalLobeLength [μm]
AvgBasalLobeWidth [μm]
AvgEquatorLobeWidth [μm]
MinCoreWidth [μm]
MaxCoreWidth [μm]
Col-0
iqd5-1
iqd5-2

B

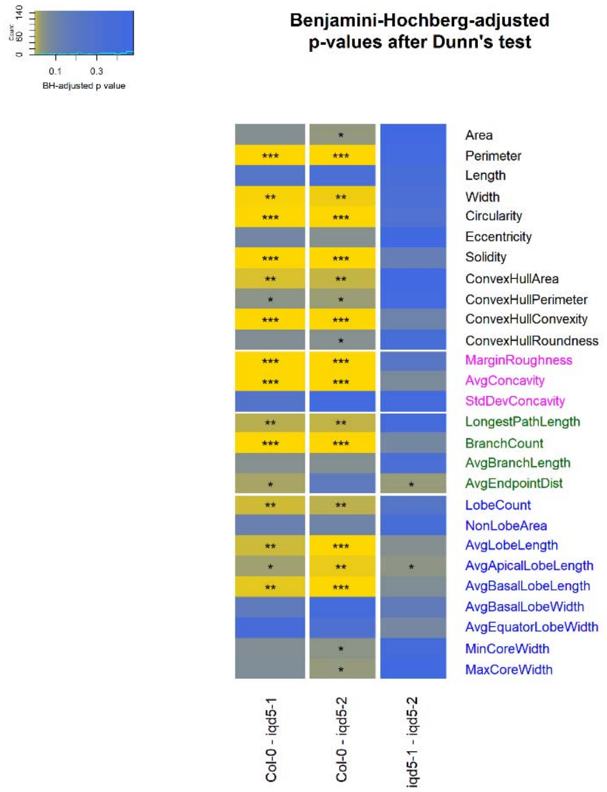

Supplementary Fig. S9.
Quantification and statistical analysis of PC shape in true leaves. PC shape features in the third and fifth true leaves of wild type and *iqd5* mutants. Violin plots of all 27 features quantified with PaCeQuant (A). Statistical analysis from pairwise comparison between the analyzed genotypes (B). Shown are Benjamini-Hochberg-adjusted p-values after Dunn‘s pairwise test. Blue colors represent p-values close to 1, yellow colors represent values close to 0. Stars indicate statistically significant differences (* padj < 0.05, ** padj < 0.01, *** padj < 0.005).

Supplementary Fig. S10.

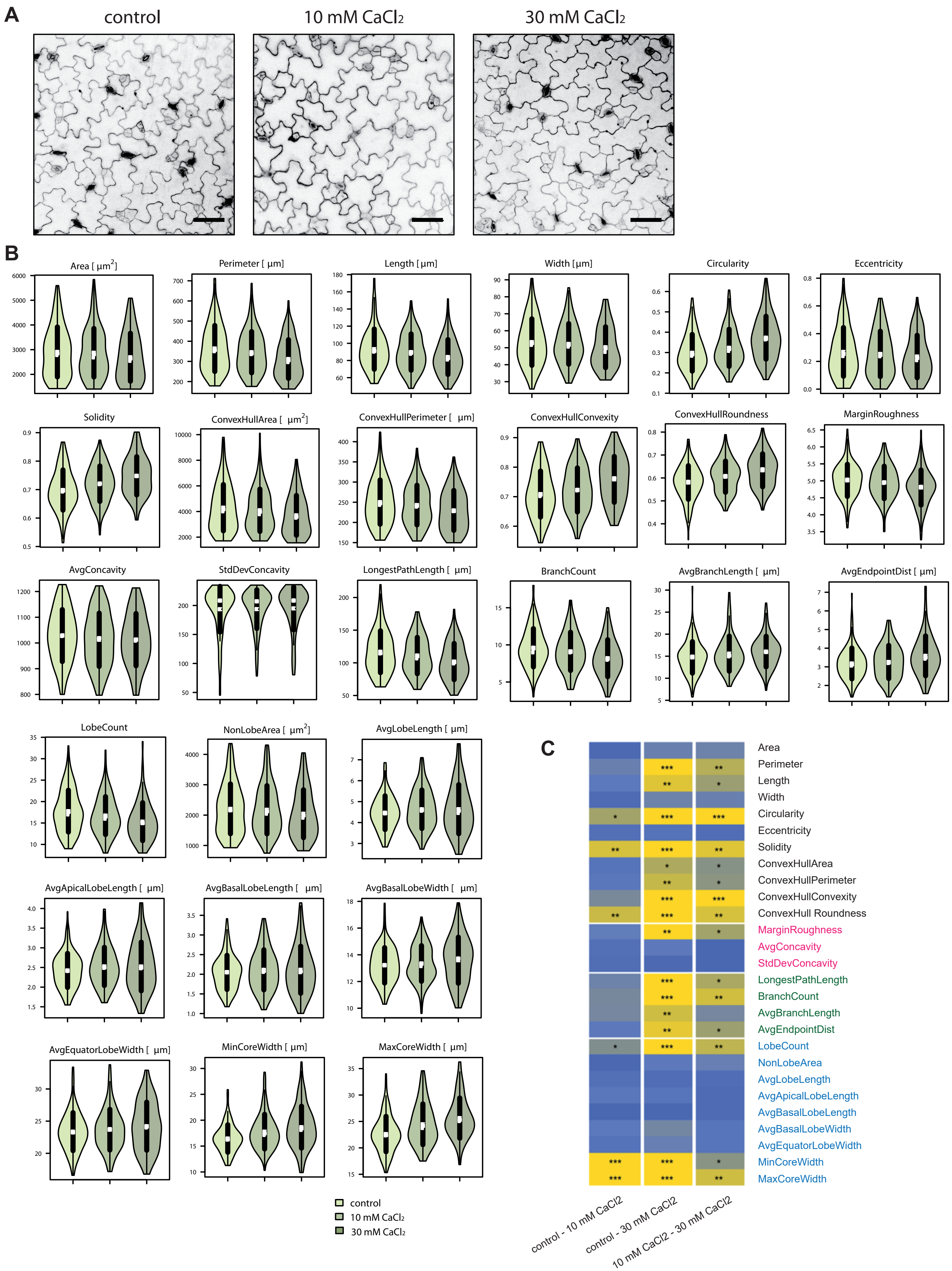

Supplementary Fig. S10.
Calcium-dependent changes in PC shape. A) Representative images of epidermis cells in seedlings 5 DAG grown on control media or on supplemented with 10 or 30 mM $CaCl_2$. B) Violin plots of all 27 shape features quantified with PaCeQuant. C) Statistical analysis from pairwise comparisons. Blue colors represent p-values close to 1, yellow colors represent values close to 0. Stars indicate statistically significant differences (*padj<0.05, **pqdj<0.01, ***padj<0.005).

Supplementary Fig. S11

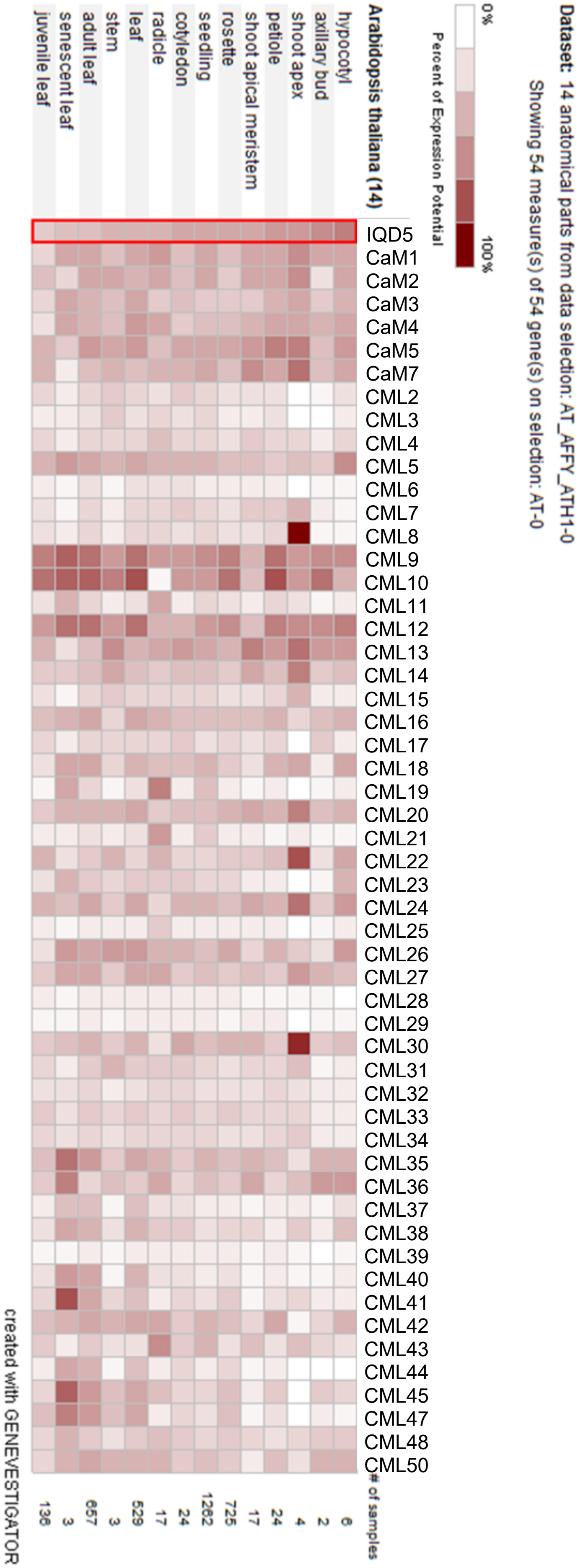

Supplementary Fig. S11.
In silico expression analysis of *IQD5,* and *CaM* and *CML* genes using Genevestigator (Zimmermann et al., 2014, BioData Mining, 7:18). Note that *CaM2/3/5* and *CaM1/4* encode for identical proteins. No expression information is available for *CaM6, CML1, CML46*, and *CML49*.
